# DNA Barcoding the Native Flowering Plants and Conifers of Wales

**DOI:** 10.1371/journal.pone.0037945

**Published:** 2012-06-06

**Authors:** Natasha de Vere, Tim C. G. Rich, Col R. Ford, Sarah A. Trinder, Charlotte Long, Chris W. Moore, Danielle Satterthwaite, Helena Davies, Joel Allainguillaume, Sandra Ronca, Tatiana Tatarinova, Hannah Garbett, Kevin Walker, Mike J. Wilkinson

**Affiliations:** 1 National Botanic Garden of Wales, Llanarthne, United Kingdom; 2 Department of Biodiversity and Systematic Biology, National Museum Wales, Cardiff, United Kingdom; 3 Department of Applied Sciences, University of the West of England, Bristol, United Kingdom; 4 Institute of Biological, Environmental and Rural Sciences, Aberystwyth University, Aberystwyth, United Kingdom; 5 Faculty of Advanced Technology, University of Glamorgan, Pontypridd, United Kingdom; 6 Botanical Society of the British Isles, Harrogate, United Kingdom; Biodiversity Insitute of Ontario - University of Guelph, Canada

## Abstract

We present the first national DNA barcode resource that covers the native flowering plants and conifers for the nation of Wales (1143 species). Using the plant DNA barcode markers *rbcL* and *matK*, we have assembled 97.7% coverage for *rbcL*, 90.2% for *matK*, and a dual-locus barcode for 89.7% of the native Welsh flora. We have sampled multiple individuals for each species, resulting in 3304 *rbcL* and 2419 *matK* sequences. The majority of our samples (85%) are from DNA extracted from herbarium specimens. Recoverability of DNA barcodes is lower using herbarium specimens, compared to freshly collected material, mostly due to lower amplification success, but this is balanced by the increased efficiency of sampling species that have already been collected, identified, and verified by taxonomic experts. The effectiveness of the DNA barcodes for identification (level of discrimination) is assessed using four approaches: the presence of a barcode gap (using pairwise and multiple alignments), formation of monophyletic groups using Neighbour-Joining trees, and sequence similarity in BLASTn searches. These approaches yield similar results, providing relative discrimination levels of 69.4 to 74.9% of all species and 98.6 to 99.8% of genera using both markers. Species discrimination can be further improved using spatially explicit sampling. Mean species discrimination using barcode gap analysis (with a multiple alignment) is 81.6% within 10×10 km squares and 93.3% for 2×2 km squares. Our database of DNA barcodes for Welsh native flowering plants and conifers represents the most complete coverage of any national flora, and offers a valuable platform for a wide range of applications that require accurate species identification.

## Introduction

Identification of plant species is of critical importance in conserving and utilising biodiversity, but this may be hindered by a lack of taxonomic expertise [1]. Other than identifying whole plants, it is also sometimes useful to be able to identify species from material such as roots, seeds, pollen or in mixtures of plants sampled from the air, soil or water, although this may be difficult or impossible using traditional morphological approaches [2]. A wide range of molecular techniques have been used to overcome this, but a growing desire for harmonisation and increased efficiency has led to a global DNA barcoding initiative to standardise molecular identifications using internationally agreed protocols and regions of DNA [2]–[4].

Following the evaluation of several candidate loci, the Plant Working Group (PWG) of the Consortium for the Barcoding of Life (CBOL) recommended that sections of two plastid genes, *rbcL* and *matK*, be adopted as the standard plant DNA barcodes, with the recognition that supplementary markers may be required [2]. Several studies have evaluated the utility of plant DNA barcodes in a taxonomic and floristic context, using these markers and others [5]–[15] and a variety of applications have been developed that show the wide potential for plant DNA barcoding. For instance, barcoding strategies have been deployed for the verification of plant products ranging from medicinal plants [16]–[18] to kitchen spices [19], berries [20], olive oil [21], tea [22] and characterisation of the plant origins of honey [23]. Ecological applications have been equally diverse including the identification of invasive species [24]–[26], characterisation of below-ground plant diversity using roots [27] and reconstruction of past vegetation and climate from plant remains in the soil [28]. Sequences obtained in the context of DNA barcoding have been used to create phylogenetic trees for use in phylogenetic community ecology [5], [29].

Use of DNA barcoding as an identification tool is dependent on the creation of high quality reference databases of sequences. Access to appropriate taxonomic expertise and an ability to utilise the vast resource of specimens already available in herbaria are key elements in achieving this [30]. DNA barcoding is particularly useful within a regional floristic context [3]. Many ecological and conservation-based DNA barcoding applications apply within a floristic context and the assembly of floristic DNA barcode databases allows this source of information to be combined with other datasets such as plant distribution and abundance records, habitat data and conservation priorities. A floristic approach allows potentially higher levels of species discrimination, as a geographically bounded sampling will usually contain fewer closely related species than a comprehensive taxonomic treatment [2], [3].

Here we present the creation of a DNA barcode database for all of the native and archaeophyte (species naturalised before 1500 AD) [31] flowering plants and conifers for the nation of Wales. Wales is an ideal exemplar to illustrate the potential of plant DNA barcoding in a floristic context. It is a small country (22,000 sq km) with a native and archaeophyte seed plant flora of 1143 species contained within 455 genera, 95 families and 34 orders [32], [33]. There is a long tradition of botanical recording, which means the flora is well studied and its national herbarium contains a comprehensive collection of the species, including many recent accessions. Taxonomic expertise is available for the entire species assemblage. Numerous national datasets are also available describing features such as plant distribution and habitat preferences that can be usefully combined with the DNA barcode database [34]–[36].

In compiling the barcode database for the 1143 species of Wales, we sampled 4272 specimens to ensure coverage of more than one individual per species. Of these, 3637 specimens came from the Welsh National Herbarium (NMW) and a further 635 samples were collected fresh from sites throughout Wales. All individuals were amplified and sequenced using the DNA barcode regions of *rbcL* and *matK* [2]. Freshly collected leaf samples were accompanied by herbarium vouchers and all successfully DNA barcoded specimens have full collection data and scans of their herbarium vouchers. The resulting sequences were checked rigorously to ensure the DNA barcodes are as accurate as possible. Multiple individuals for each species were compared against each other and to additional sequences downloaded from the Barcode of Life Database (BOLD) [37] and GenBank.

We examined recoverability of DNA barcodes from herbarium versus freshly collected material and the effect of herbarium specimen age. We assessed the ability of our DNA barcodes to identify species by examining for the presence of barcode gaps (using pairwise and multiple alignments) [2], monophyletic groups using Neighbour-Joining (NJ) trees and similarity approaches using BLASTn [5], [38]. The database was further tested using 1346 *rbcL* and 1380 *matK* sequences downloaded from GenBank and used as query sequences. Finally, we assessed the scope for improving species discrimination by looking at resolution at different spatial scales. The number of plant species that could be identified within 10×10 km squares was investigated for the whole of Wales and for three regional areas, levels of discrimination were examined within 2×2 km squares.

This DNA barcode initiative represents the most comprehensive sampling of any national flora to date, both in terms of the proportion of the flora covered, and the number of individuals sequenced per species. It also represents the largest DNA barcode dataset to utilise herbarium material.

## Results

### Recoverability

A total of 5,723 barcode sequences were obtained for 3378 individuals of the 1143 native and archaeophyte flowering plants and conifers of Wales (Dataset S1). These include 3304 individual sequences covering 97.7% of species for *rbcL*, and 2419 sequences representing 90.2% of species for *matK* (Tables 1 & 2). For *rbcL*, 91.1% of species are represented by more than one individual and 71.2% for *matK*. In total, dual DNA barcodes comprising both *rbcL* and *matK* were obtained for 89.7% of the Welsh flora (Table 1).

**Table 1:** Summary statistics for the 1143 species of Welsh flora DNA barcoded using *rbcL* and *matK.*

	*rbcL*	*matK*	*rbcL* & *matk*
Number of species successfully DNA barcoded (%)	1117 (97.7)	1031 (90.2)	1025 (89.7)
Number of species with more than 1 individual DNA barcoded (%)	1041 (91.1)	814 (71.2)	808 (70.7)
Mean (SD) number of individuals DNA barcoded per species	2.89 (1.1)	2.1 (1.1)	2.1 (1.1)
Mode of individuals DNA barcoded per species	3	3	3
Range of individuals DNA barcoded per species	1–9	1–8	1–8

**Table 2:** Recoverability of DNA barcodes from herbarium and fresh material for *rbcL* and *matK.*

	*rbcL*			*matK*		
	overall	herbarium	fresh	overall	herbarium	fresh
Number of samples collected	4272	3637	635	4272	3637	635
Number successfully DNA barcoded (%)	3304 (77.3)	2705 (74.4)	599 (94.3)	2419 (56.6)	1917 (52.7)	502 (79.1)
Amplification failure (%)	605 (14.2)	591 (16.2)	15 (2.4)	1427 (33.4)	1367 (37.6)	61 (9.6)
Sequencing failure (%)	157 (3.7)	141 (3.9)	15 (2.4)	265 (6.2)	217 (6.0)	47 (7.4)
Incorrect sequence (%)	206 (4.8)	200 (5.5)	6 (0.9)	161 (3.8)	136 (3.7)	25 (3.9)

Recoverability of DNA barcodes for *rbcL* was high overall (77.3%) but this varied according to the nature of the source material, with sequences recovered from 94.3% of fresh samples and 74.4% of herbarium specimens (Table 2). *matK* proved more problematic than *rbcL*, with an overall recoverability of 56.6% (79.1% from fresh samples and 52.7% from herbarium material).

The lower performance using herbarium material was mostly due to lower amplification success (Table 2). For *rbcL*, amplification failure was 2.4% for fresh material and 16.2% for herbarium material. For *matK*, amplification failure was 9.6% for fresh material and 37.6% for herbarium specimens. There is also an interaction between *matK* primer specificity and material type. For fresh samples, 79.1% DNA barcoding success was achieved using 5 *matK* primer combinations. Herbarium material required greater use of order specific primers, with 23 primer combinations used to achieve a success of 52.7% (Dataset S2). Sequencing failure is higher for *matK* relative to *rbcL* but there are not marked differences between fresh and herbarium material.

In addition to amplification or sequencing failure, the other reason for not obtaining a DNA barcode was incorrect sequences being found during data processing and checking. Sources of error include sample mis-labelling, either on the herbarium specimen or when the sample was removed and processed in the herbarium or lab, contamination of samples, either in the lab or herbarium, and mistakes during data processing such as incorrect forward and reverse sequences put together during contig assembly. The reasons for incorrect sequences were not always clear but the overall level was relatively low (4.8% for *rbcL* and 3.8% for *matK*) (Table 2). Herbarium specimens were double-checked in cases where DNA samples did not appear to match the herbarium voucher. The level of herbarium specimens that were found to be incorrect was just 0.2% (8 out of 3637 samples). In most cases this was due to mixed samples being mounted on herbarium sheets. More rarely, contamination of samples was found, for example from algae dried on the leaves of aquatic species on herbarium sheets. For *matK* levels of incorrect sequences were comparable using fresh (3.9%) and herbarium material (3.7%). Levels of incorrect sequences were significantly higher for *rbcL* at 4.8% for herbarium material but just 0.9% when fresh specimens were used (chi-squared test with Yate's correction: chi^2^ = 18.0, p = <0.0001).

Sequence quality was acceptable overall (Table 3 & Dataset S2). Levels of bidirectional reads were high, averaging from 81.0–90.1% for *rbcL* and *matK* (from fresh and herbarium material). The mean percentage of high quality bases within the sequences (defined as a QV score greater than 30) ranged from 91.0–97.2% for both markers using herbarium and fresh material. Levels of gaps and substitutions when aligning the forward and reverse reads were low (0.03–0.1%). Using the CBOL Plant Working Group [2] criteria for high quality sequences (see Methods and Analysis), *rbcL* performs well with 63.4% of herbarium specimens yielding high quality sequences and 80.2% of fresh specimens. *matK* does not perform well using these criteria, with 33.3% of fresh and 29.8% of herbarium specimens providing high quality sequences. This low figure is due to the percentage of low quality bases being greater than 1% in many sequences, the mean being 2.0% for fresh samples and 2.6% for herbarium material.

**Table 3:** Sequence quality of DNA barcodes from herbarium and fresh material for *rbcL* and *matK.*

	*rbcL*			*matK*		
	overall	herbarium	fresh	overall	herbarium	fresh
Mean (SD) QV of sequences	55.5 (5.1)	55.2 (5.4)	56.6 (3.9)	50.2 (5.4)	49.8 (5.5)	51.6 (4.7)
Mean (SD) overlap of sequences (%)	81.3 (7.2)	81.0 (7.1)	82.3 (7.3)	83.7 (16.7)	82.0 (17.8)	90.1 (9.7)
Mean (SD) of high quality bases: QV>30 (%)	96.3 (4.3)	96.1 (4.5)	97.2 (3.6)	91.4 (7.1)	91.0 (7.3)	92.9 (5.7)
Mean (SD) of low quality bases: QV<20 (%)	1.3 (1.6)	1.4 (1.7)	1.1 (1.1)	2.5 (2.4)	2.6 (2.5)	2.0 (1.8)
Mean (SD) of internal gaps when aligning F & R read (%)	0.1 (0.2)	0.1 (0.2)	0.1 (0.1)	0.1 (0.3)	0.1 (0.3)	0.1 (0.2)
Mean (SD) of substitutions when aligning F & R read (%)	0.1 (0.3)	0.1 (0.3)	0.03 (0.3)	0.2 (0.5)	0.2 (0.5)	0.1 (0.3)
High quality sequences using CBOL PWG criteria (%)	66.4	63.4	80.2	30.5	29.8	33.3

For a complete list of quality values for each DNA barcode see Dataset S2. For CBOL PWG criteria see Methods and Analysis.

**Figure 1:**
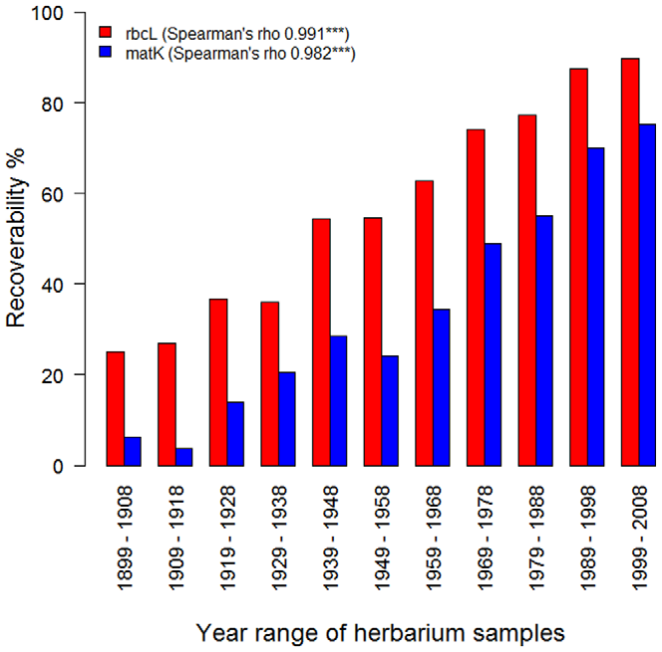
Effect of herbarium specimen age on recoverability (%). Using 3607 herbarium specimens ranging in age from 1899–2008, samples were divided into 11 age classes and Spearman rank correlation used to test for a relationship between age class and recoverability (%) using the DNA barcode loci, *rbcL* and *matK.*

The oldest herbarium sample successfully DNA barcoded was from 1868 but older samples showed a distinct reduction in recoverability (Fig. 1). For both *rbcL* and *matK* a highly significant, negative relationship was seen between sequencing success and herbarium specimen age (Spearman's rank correlation coefficient for *rbcL* was 0.991, p = <0.001, for *matK* 0.982, p = <0.001).

**Figure 2:**
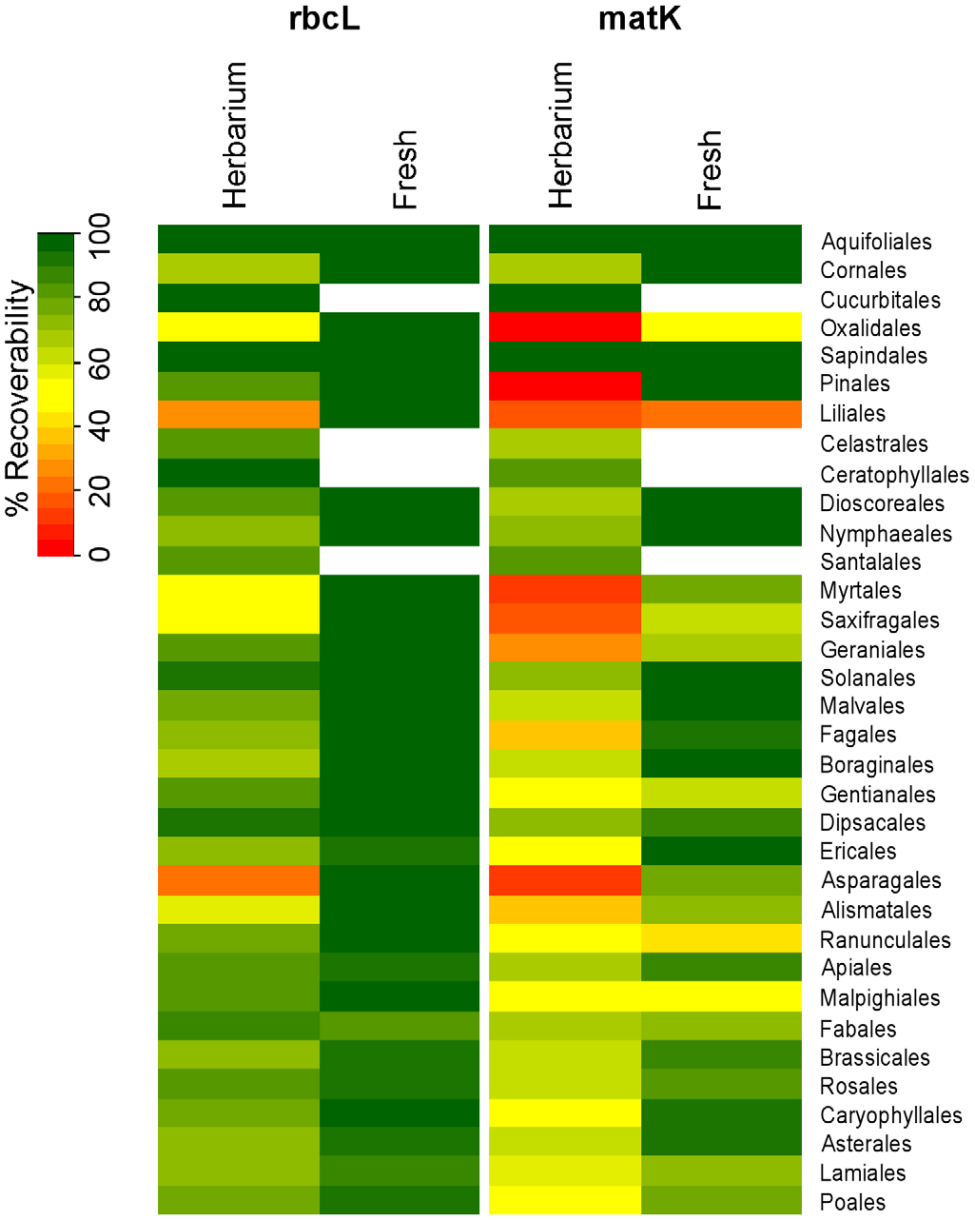
Recoverability of the orders of flowering plants and conifers found within Wales using herbarium and fresh material. Recoverability (%) of *rbcL* and *matK* across the 34 orders of seed plants found within the Welsh flora. Results are based on 3637 herbarium and 635 freshly collected specimens. White cells denote orders for which fresh specimens were not collected.

Recoverability varied across the orders of flowering plants and conifers for the two markers and depended on the source material. Freshly collected material had higher levels of success and worked more consistently across all orders (Fig. 2). Some orders were distinctly harder to DNA barcode using herbarium compared to fresh material; Oxalidales, Liliales, Myrtales, Saxifragales and Asparagales had a recoverability of less than 50% for both *rbcL* and *matK* when using DNA from herbarium specimens.

*rbcL* worked very well using fresh material and still performed acceptably for most orders when herbarium specimens were used. For *matK*, freshly collected material generally worked better than herbarium specimens but some orders sequenced poorly for both herbarium and fresh material. Oxalidales, Liliales and Ranunculales have recoverability below 50% for herbarium and fresh material, suggesting lower primer performance (Fig. 2).

### Interspecific and intraspecific divergence

*matK* showed higher levels of genetic divergence compared to *rbcL*. Mean interspecific divergence for *matK* was 0.017 (SD 0.023), compared to 0.006 (SD 0.007) for *rbcL* (Table 4). Levels of intraspecific divergence were also higher for *matK*, but for both markers levels of within species variance for Wales were low; just 6.3% of species exhibited any intraspecific variance for *rbcL* and 16.7% for *matK* (Table 4).

**Table 4:** Interspecific and intraspecific divergence for *rbcL* and *matK.*

	*rbcL*	*matK*
Mean interspecific divergence (SD)	0.0063 (0.0069)	0.0174 (0.0231)
Mean intraspecific divergence for all individuals (SD)	0.0001 (0.0005)	0.0003 (0.0009)
Mean intraspecific divergence theta (SD)	0.0001 (0.0006)	0.0004 (0.0011)
Mean coalescent depth (SD)	0.0001 (0.0006)	0.0004 (0.0012)
Proportion of species showing intraspecific variation (%)	66/1041 (6.3)	136/814 (16.7)

Levels of divergence were determined using uncorrected p-distances calculated from multiple alignments. Interspecific divergence includes only those genera with more than 1 species per genus (*rbcL* 199, *matK* 184 genera). Intraspecific divergence includes only those species with more than 1 individual sampled per species (*rbcL* 1041, *matK* 814 species).

### Discrimination

**Figure 3:**
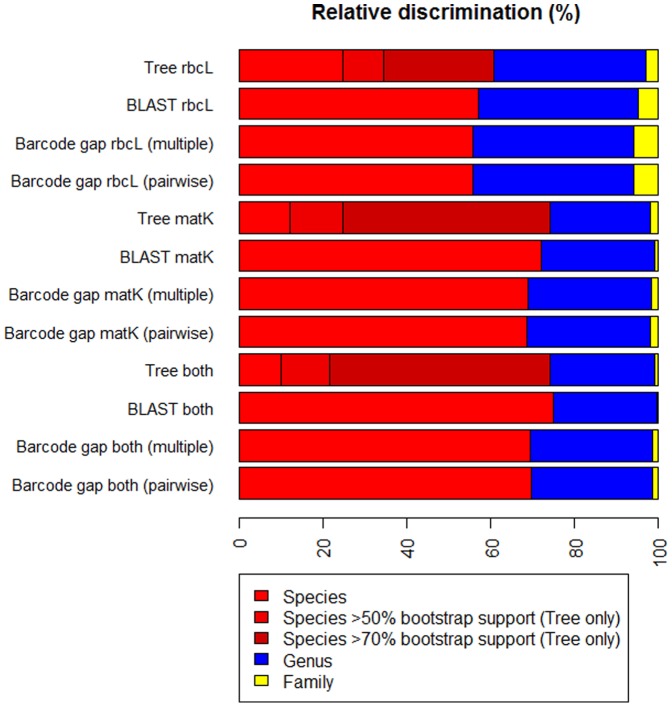
Ability of the DNA barcode markers *rbcL* and *matK* to discriminate the Welsh flora. Discrimination (%) at species, genus and family level for *rbcL*, *matK* and both markers combined using monophyletic groups in Neighbour-Joining trees (Tree), BLASTn searches (BLAST) and barcode gap analysis using pairwise (Barcode gap pairwise) and multiple alignments (Barcode gap multiple). Species level discrimination for monophyletic groups in Neighbour-Joining trees is divided into bootstrap support values of ‘any’, >50% and >70% based on 1000 bootstrap replicates. Discrimination uses 808 species for which multiple individuals were DNA barcoded for both *rbcL* and *matK.* Species with single sequences were included in the analyses as sources of discrimination failure. For a complete list of which species can be discriminated using the different methods see Dataset S3.

In order to compare across markers and methods of discrimination, we used a dataset of 808 species for which multiple individuals were sequenced for both *rbcL* and *matK* to provide a measure of relative discrimination. Species represented by single sequences were included in the analyses to serve as sources of discrimination failure (decoys). The four approaches for measuring discrimination success, barcode gap (pairwise and multiple alignment), monophyletic groups in NJ trees and BLASTn, provided broadly similar results (Fig. 3 & Dataset S3). Relative discrimination across all four methods was highest using a combined *rbcL* and *matK* matrix, the different methods provided a range of 69.4–74.9% discrimination at the species level and 98.6–99.8% discrimination to genus. *matK* performed well on its own, with 68.7–74.1% of species and 98.0–99.1% of genera discriminated. This compares with 55.8–60.9% of species discriminated with *rbcL* and 94.3–97.2% of genera.

Of the four methods the NJ tree tended to discriminate most species but this decreased as more stringent levels of bootstrap support were used. There was higher bootstrap support for the monophyletic groups generated using the combined and *matK* NJ trees than for those produced using *rbcL*. For the combined *rbcL* and *matK* tree, 86.5% of monophyletic species had >50% support and 70.8% had >70% support. These levels were lower for *rbcL*, with 59.3% at >50% and 43.3% at >70%. BLAST was next best for discrimination, followed by barcode gap analyses. The use of a pairwise versus multiple alignment to calculate uncorrected p-distances made almost no difference to the species that could be discriminated (Dataset S3). For *rbcL* 55.8% of species could be discriminated when a pairwise or multiple alignment was used. Species discrimination for *matK* was 68.7% using a pairwise alignment and 68.8% using a multiple alignment. When markers were combined 69.7% of species were discriminated with the pairwise alignment and 69.4% with the multiple alignment.

**Figure 4:**
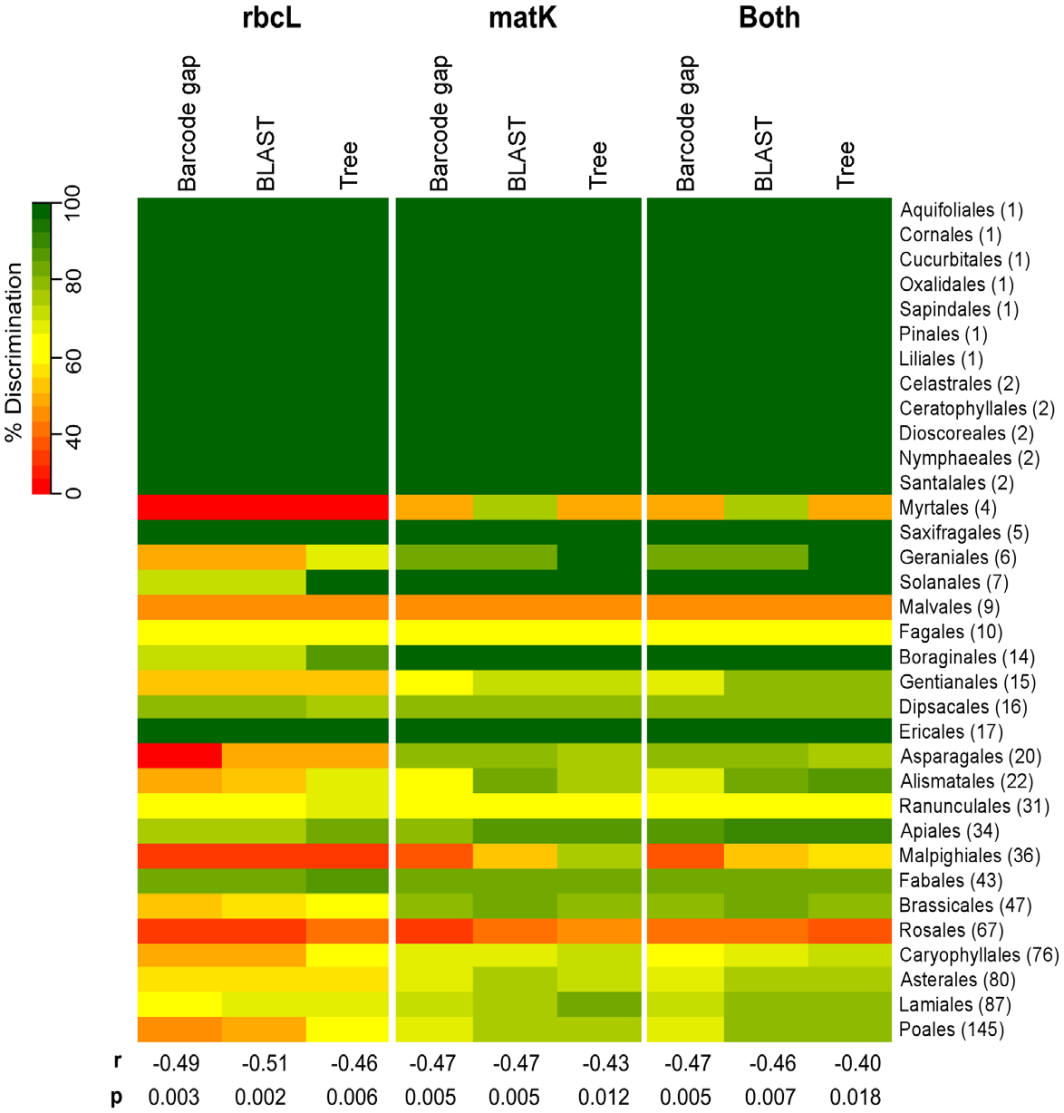
Species discrimination for the orders of flowering plants and conifers found within Wales. Species discrimination (%) for *rbcL*, *matK* and both combined across the 34 orders of flowering plants and conifers found within the Welsh flora. Discrimination is assessed using three methods; barcode gap using multiple alignments (Barcode gap), monophyletic groups in Neighbour-Joining trees (Tree) and BLASTn searches (BLAST). To allow for comparison across the markers and methods 808 species for which multiple individuals were sequenced for both *rbcL* and *matK* were used, but species with single sequences were included as a source of discrimination failure. The number of species per order in the Welsh flora (out of the 808) is shown in brackets next to the order name. Pearson correlation coefficients and associated p-values for the relationship between the number of species per order and % species discrimination success are shown.

Levels of relative discrimination varied across the orders of flowering plants and conifers (Fig. 4). These were significantly negatively correlated with the number of species DNA barcoded within the order, with Pearson correlation coefficients ranging from −0.40 to −0.51 (p-values 0.018 to 0.002) using the different markers and methods of discrimination. Some orders had higher levels of discrimination than expected given the number of species they contain (Boraginales and Ericales) whilst others had lower levels than expected (Myrtales, Malvales, Malpighiales and Rosales).

### Testing discrimination using DNA sequences from GenBank

GenBank sequences were used to provide an additional test of the discrimination ability of our DNA barcodes. GenBank data provide an effective test as sequences from GenBank comprise different length fragments of *rbcL* and *matK* from species collected from a broader geographic coverage than had been sampled for the Wales DNA barcode database. Furthermore, sequence quality is not subject to the higher standards stipulated for DNA barcoding and so may be prone to exaggerate the level of variability between samples.

We recovered 2726 sequences from GenBank that correspond to species found within the Welsh flora (Table S3). The *rbcL* dataset included 1346 sequences covering 592 species, *matK* comprised 1380 sequences that covered 533 species (Table 5). BLASTn results using the GenBank data showed similar levels of discrimination to those obtained using our data. Some 57.4% of *rbcL* sequences allowed identification to species and 93.2% to genus; for *matK*, 66.6% of sequences were identified to species and 95.4% to genus (Table 5).

**Table 5:** Testing the ability of the Welsh flora DNA barcode database to identify sequences downloaded from GenBank.

		n	Species (%)	Genus (%)	Family (%)	Failed (%)
***rbcL***	Total number of sequences identified	1346	773 (57.4)	1255 (93.2)	1338 (99.4)	8 (0.6)
	Number of species identified for at least one sequence	592	346 (58.4)	557 (94.1)	591 (99.8)	1 (0.2)
	Number of species identified with all sequences available for that species	592	299 (50.7)	527(89)	584 (98.6)	8 (1.4)
***matK***	Number of sequences identified	1380	919 (66.6)	1317 (95.4)	1368 (99.1)	12 (0.9)
	Number of species identified for at least one sequence	533	383 (71.9)	514 (96.4)	529 (99.2)	4 (0.8)
	Number of species identified with all sequences available for that species	533	347 (65.1)	490 (91.9)	521 (97.7)	12 (2.3)

*rbcL* and *matK* sequences downloaded from GenBank were queried against the Welsh flora database for *rbcL* and *matK* using a BLASTn search.

### Discrimination at different spatial scales

**Figure 5:**
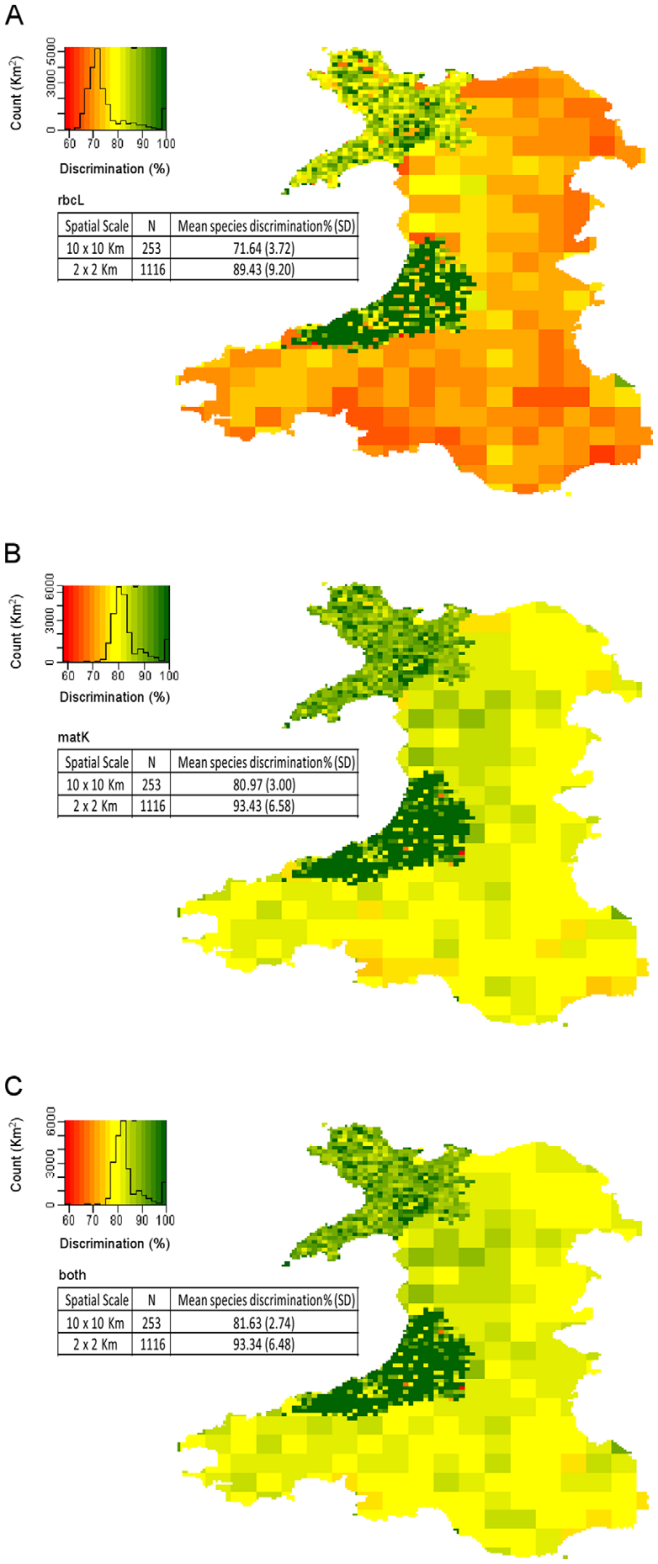
Species discrimination success (%) at different spatial scales across Wales. Species discrimination for Wales at the 10×10 km level and for 3 vice-counties at the 2×2 km level for A) *rbcL* B) *matK* and C) combined. This uses 891,756 plant species records from the Botanical Society of the British Isles. Species discrimination for each square is determined by taking the species list for that square and conducting barcode gap analysis (using multiple alignments).

Reducing the spatial scale from the whole of Wales to smaller units of area improved the potential for species-level diagnosis by reducing the total number of candidate species being compared. Examining for the presence of a barcode gap (using a multiple alignment) at the 10×10 km level provided a mean species discrimination for *rbcL* of 71.6% (SD 3.7), *matK* of 81.0% (SD 3.0) and 81.6% (SD 2.7) for the combined markers. This further improves at the 2×2 km level to 89.4% (SD 9.2) for *rbcL*, 93.4% (SD 6.6) for *matK* and 93.3% (SD 6.5) for the combined markers (Fig. 5).

## Discussion

The creation of a DNA barcode database for a nation's flora provides a powerful platform for a broad range of applications that are reliant upon large-scale species identification. Our database of DNA barcodes for Welsh native flowering plants and conifers represents the most complete sampling of any national flora to date. It also represents the largest sampling of herbarium material to date.

Both *rbcL* and *matK* perform better using DNA extracted from freshly collected material compared to herbarium specimens. Amplification and sequencing success and primer universality for *rbcL* is good, but the lower recoverability and universality of *matK* using herbarium specimens increases the laboratory time required to assemble a comprehensive database. *matK* also has lower sequence quality compared to *rbcL* which increases the requirement for manual editing. Using the CBOL Plant Working Group [2] criteria for high quality sequences yields a low sequence quality for *matK* in this study. This is caused however by a slightly higher percentage of low quality bases relative to their 1% threshold. We recommend that users of DNA barcode databases examine actual quality scores rather than rely on threshold levels whose cut off values can be difficult to assign in an objective way.

The increase in laboratory and data processing time when herbarium material is used however is far less than the time required to collect, identify and verify new specimens from the wild. Herbaria provide a readily accessible stock of plant material that can be rapidly sampled. More importantly, DNA barcoding herbarium specimens effectively captures the years of taxonomic expertise that have gone into the creation of the herbarium resource and translates this into an accessible tool for DNA-based identification [39].

Nevertheless, use of herbarium specimens does add extra considerations compared to fresh material. Herbarium specimens often require more attempts at amplification with more primer combinations. This potentially increases the possibility of obtaining incorrect sequences through increased chances of samples becoming mixed up or contaminated. For *matK* this does not appear to be the case but for *rbcL* levels of incorrect sequences were higher using herbarium material than freshly collected specimens, most likely due to its greater primer universality and ease of amplification. Some types of specimen also required greater caution; for example contamination of aquatic species with algae was difficult to detect when sampling herbarium specimens. Some orders of flowering plants do not sequence well using herbarium material for either *rbcL* or *matK*, most notably Oxalidales, Liliales, Myrtales, Saxifragales and Asparagales, and the collection of fresh material is recommended for these orders. Younger herbarium specimens work significantly better than older specimens, so DNA barcode campaigns should focus on younger material. Other studies have generally found no link or only a weak correlation between herbarium specimen age and DNA recoverability, however, these tend to focus on a narrower taxonomic range of species and use substantially smaller sample sizes [40]–[42]. This study which has tested 3637 herbarium specimens across 34 orders, with the oldest sample successfully DNA barcoded from 1868, represents the most comprehensive use of herbarium material for DNA extraction to date.

The use of herbarium material in this study illustrates the substantial value of the world's herbaria in capturing taxonomic expertise. With advances in GIS, digital imaging and DNA technologies it is timely to consider a new gold-standard for herbarium specimens, where every specimen collected routinely includes GPS location, digital photographs and a silica gel collection. This would make a project such as this one more straightforward and powerful in the future, especially since future projects are likely to involve the sequencing of entire genomes rather than a limited number of markers.

For all DNA barcodes thorough checks need to be made after sequencing to identify incorrect sequences. The sequencing of multiple individuals per species is of critical importance as it allows comparisons between the sequences to be made. This can be supplemented by comparing the sequences against those of that species available in BOLD [37] and GenBank. Whilst this process easily identifies incorrect sequences which are taxonomically distant from the target species, it is much harder to identify incorrect sequences from closely related species. Laboratory procedures can help to minimise errors such as not processing congeneric species in adjacent wells, but in some cases only increased sample sizes per species will reveal errors.

DNA barcoding of plants has a broad range of uses, from ecological forensics and understanding plant community structure to commercial applications [43]. Each of these applications places different requirements on the markers to be used. Many applications of DNA barcoding are likely to focus on poorer quality template, such as DNA from faecal material, stomach contents or processed samples [3]. Our results using herbarium material illustrate that markers can perform differently depending on the source material used; for example, the greater need for *matK* primer specificity using DNA from herbarium specimens. DNA barcoding applications are also increasingly making use of next-generation sequencing approaches, which enable the analysis of mixtures of samples. This requires consistent amplification across the different species within the mixture and currently places limits on the length of fragment that can be analysed. The ease of amplification, universality and c.600 bp size makes *rbcL* ideal for a wide range of applications. *matK* is much more difficult to use but it does provide greater levels of interspecific divergence compared to *rbcL.* We suggest that the future choice of markers for DNA barcoding needs to consider not only the ability to create a reference database of DNA barcodes, but also the range of material and analysis methods likely to be used for DNA barcoding applications.

The species level resolution of 69.4–74.9% using *rbcL* and *matK* for the whole of Wales is comparable to some other studies using the same markers, although the number of species we have sampled is greater [3]. Differences in the number of species examined, sample size and methods to measure discrimination make comparisons with other studies difficult, but discrimination figures of c.70% are often found when a broad taxonomic coverage has been used [3]. The number of individuals and species we have DNA barcoded is similar in size to a recent study by the China Plant BOL Group [15]. Comparing our dataset of 808 species for which we have multiple individuals DNA barcoded for both markers with their dataset of 765 species with multiple individuals, our levels of species discrimination are substantially higher. Our barcode gap analysis using pairwise alignments is the same as their ‘PWG-Distance’ metric. The China Plant BOL Group report species level discrimination of 26% for *rbcL*, 46% for *matK* and 50% for both markers, compared to our results of 55.8% for *rbcL*, 68.7% for *matK* and 69.7% for both [15]. The difference in results can be attributed to the much greater number of closely related species included in their study. The native Welsh flora contains 455 genera compared to the China Plant BOL Group sample of 141 genera [15]. This clearly demonstrates the large differences in discriminatory power of DNA barcode campaigns in parts of the world with greatly differing floristic diversity.

Discrimination success can be considerably improved by concentrating on a geographically defined set of species. Using plant distribution data to reduce the number of likely species substantially improved potential levels of discrimination at smaller spatial scales, allowing mean species level discrimination of 81.6% at 10×10 km and 93.3% at the 2×2 km level, using both markers. This does not provide complete certainty of an identification, since it is only as good as the species list for that area. In well-characterised countries such as Wales, however, this provides a useful mechanism for increasing discrimination without the need for additional sequencing. It is akin to traditional approaches where a botanist will typically look at the distribution of the species, its rarity and its habitat to provide support for their identification based on morphology. A decision framework can be developed where the possible identity of the species using sequence information is qualified with additional data to assign a species identification with an associated confidence level. Location, along with information on habitat type, time of year and abundance can all be used to improve and verify species identification by reducing the number of candidate species. Morphological characters can be used as ‘tie-breakers’ for species that share a DNA barcode.

The DNA barcode database presented here provides a powerful research and development platform at a national scale. A broad range of applications can be developed that rely on the fact that the majority of Welsh species can be identified to species level using a DNA barcoding approach. Beyond this, it provides a stock of pre-existing barcodes that can be assimilated into similar databases created for other regions, especially those within Europe. The 1143 species of Wales represents 76% of the UK flora [33] and contributes to the European flora. This, coupled with the provision of methodological procedures, especially in the use of herbarium material, eases the path for similar projects to be completed in other parts of globe.

## Methods and Analysis

### Sample collection

The Welsh flora comprises 1143 native and archaeophyte species of flowering plants and conifers (455 genera, 95 families and 34 orders) using aggregate species for the apomictic groups of *Rubus*, *Taraxacum* and *Hieracium* [32], [33]. In total 4272 individuals were sampled; 3637 from herbarium specimens (NMW) and 635 from living plants collected throughout Wales. All herbarium and fresh material was verified by a taxonomic expert and herbarium vouchers made for freshly collected plants. For threatened species where the collection of a herbarium voucher was not acceptable on conservation grounds, a photograph was used to act as the voucher. For freshly collected plants all necessary permits were obtained before collection of specimens. Permission was obtained from the Countryside Council for Wales before collection of material from sites designated for nature conservation and the land manager and/or owner for private sites.

For herbarium material at least 3 herbarium specimens of each species where selected for DNA barcoding as follows: first, as typical examples of each species, preferably which had been determined by an expert in addition to the authors; second, for which small samples of tissue (approximately 2 cm^2^) could be taken without detracting from the scientific value of the specimen; third, were from geographically separate locations within Wales; and fourth, were recently collected. No type specimens or historically important material was sampled.

### DNA extraction, amplification and sequencing

Leaf material from freshly collected plants was dried in silica gel and the DNA extracted using Qiagen DNeasy kits following the manufacturer's protocol. The protocol was modified for herbarium samples to improve amplification and sequencing success. To the 400 µl of AP1 buffer was added 80 µl of DDT at 0.75 mg/ml (Melford Laboratories, UK) and 20 µl of Proteinase K at 1 mg/ml (Sigma) before disruption using a TissueLyser II (Qiagen) with 3 mm tungsten carbide beads. After disruption, incubation in the modified AP1 buffer was extended to 1 hour at 65°C. The final incubation stage with AE buffer was extended to 15 minutes.

*rbcL* amplification used a universal primer (*rbcL*a-F) in combination with one of five reverse primers (Table S1 & Dataset S2). Initial amplifications used the *rbcL*a-F and *rbcL*r590 combination, if this failed, then a different reverse primer was tried. For *matK*, a total of 23 primer combinations were used in order to maximise amplification and sequencing success (Table S2 & Dataset S2). Universal primer combinations were tested first and if these failed, order specific primers were used. The emphasis was on obtaining sequences for as many species as possible, not limiting the number of primers used or the number of amplifications attempted.

DNA was amplified in a 20 µl reaction mixture containing 10 µl of 2× Biomix (Bioline, UK), 0.4 µl (10 µM) of F and R primer, 0.8 µl of BSA at 1 mg/ml (New England Biolabs) 6.4 µl of H_2_O (Sigma) and 2 µl of template DNA. PCR cycling conditions were [95°C 2 min (95°C 30 sec, 50°C 1 min 30, 72°C 40 sec)×45 cycles, 72°C 5 min, 30°C 10 sec]. PCR products were visualized on 1% agarose gels and samples showing suitably bright bands were sent to Macrogen Europe (Amsterdam, Europe) for purification and DNA sequencing in both directions on an ABI3730X, using the same primers as used for PCR.

### Sequence editing, checking and alignment

Sequencher 4.10.1 (GeneCodes Corp) was used to trim ends (using a 25 bp window segments with >2 bp showing QV<20 were removed), remove primers and assemble contigs. Every contig was checked for base call disagreements and ambiguities and manually edited where necessary. Poor quality sequences that were not amenable to manual editing, those with low overlap (generally less than 50%) and any sequences with stop codons were removed. Quality statistics including amount of bidirectional read, mean QV of sequences, the percentage of high (QV>30) and low quality (QV<20) bases and amount of internal gaps and substitutions when aligning the forward and reverse reads were calculated for each contig and summarised for herbarium and fresh material. In addition the number of contigs meeting the criteria for high quality sequences according to the CBOL Plant Working Group [2] was determined. The CBOL PWG define high quality sequences as those in which both the forward and reverse reads should have a minimum length of 100 bp, a minimum mean QV of >30 and the post-trim lengths should be >50% of the original read length; the assembled contig should have >50% overlap in the alignment of the forward and reverse reads with <1% low-quality bases (<20 QV) and <1% internal gaps and substitutions when aligning the forward and reverse reads.

Sequences were thoroughly checked to ensure that any incorrect sequences were found. Multiple individuals for each species were compared to each other and Neighbour-Joining trees examined for misplaced species. Sequences matching the species in the Welsh flora were downloaded from BOLD [37] and GenBank and compared to our sequences to look for possible errors. Sequences are deposited in GenBank and sequences along with collection data and scans of the herbarium vouchers on BOLD (Dataset S1).

In order to compare results using different alignment methods both pairwise and multiple alignments were used. All possible global pairwise alignments were calculated using the Needleman-Wunsch algorithm and uncorrected p-distances determined for each pair using only unambiguous sequence differences [44] For the multiple alignments, *rbcL* sequences were aligned using MUSCLE v3.7 [45] with default parameters. For *matK*, transAlign v1.2 [46] was used to translate nucleotides into amino acids, align using Clustal W v2.1 and back-translate, with the resulting alignment checked and manually edited using Geneious Pro v5.4.4. After squaring the ends of the matrices, the final multiple alignment lengths were 542 bp for *rbcL* and 897 bp (with gaps) for *matK*. Uncorrected p-distances were calculated from the multiple alignment for each pairwise comparison. All alignments were carried out using the HPC Wales supercomputer cluster. A script written in Python was used to concatenate the *rbcL* and *matK* alignments into a single matrix, *rbcL* first and then *matK*.

### Recoverability

Sequencing success was assessed overall and compared across herbarium and fresh material and different orders of flowering plants and conifers. The effect of herbarium specimen age was evaluated by dividing specimens into 11 age classes from 1899 to 2008. 30 specimens successfully sequenced from 1868–1898 were not included in the analysis as the sample sizes for each age class were too small. Effect of age class on sequencing success was assessed using Spearman rank correlations.

### Interspecific and intraspecific divergence

Interspecific divergences for *rbcL* and *matK* were calculated using uncorrected p-distances (from the multiple alignments) for all genera containing more than one species per genus. Three measures of intraspecific divergence were used [6], [47], intraspecific ‘all’ is the mean uncorrected p-distance for all individuals sampled for that species. Intraspecific ‘theta’ uses only the distances between different haplotypes within a species to eliminate biases associated with uneven sampling across different species. Coalescent depth is the maximum intraspecific distance observed between individuals for that species.

### Discrimination

To compare the relative discrimination of *rbcL*, *matK* and combined, discrimination was assessed for 808 species for which *rbcL* and *matK* were both sequenced for multiple individuals per species. Species with single sequences were included in the analyses to serve as sources of discrimination failure. This was assessed both overall and for each order of flowering plants and conifers. Four approaches were used to test for discrimination between different species. Presence of a barcode gap using the methods of the CBOL Plant Working Group, but using multiple as well as pairwise alignments [2]. Formation of monophyletic groups based on Neighbour Joining trees using Kimura-2-Parameter and 1000 bootstrap replicates, with the number of monophyletic species at the level of any, >50% and >70% bootstrap support recorded for all species with multiple individuals. BLASTn searches [38] with each sequence used as both database and query.

For the barcode gap analyses, matrices of pairwise uncorrected p-distances were created from the pairwise and multiple alignments (the CBOL Plant Working Group use pairwise alignments [2]). Discrimination was considered successful if the minimum interspecific p-distance involving a species was larger than its maximum intraspecific p-distance. For the BLASTn analysis, a local database was made for the *rbcL*, *matK* and the concatenated matrix using a script written in Python and each sequence then used as a query in a BLASTn search. A sequence was considered to be correctly assigned when that species had the highest Bit-Score among all candidates. A sequence counted as failed when the correct species was either tied with another species or received a lower score [5]. The query sequence was removed from the database each time.

To assess whether level of discrimination at the order level related to the number of species within that taxonomic group we examined correlations between the number of species we had DNA barcoded in each order and the percentage discrimination achieved using the methods described above.

### Testing discrimination using DNA sequences from GenBank

*rbcL* and *matK* sequences matching the species found in the Welsh flora were downloaded from GenBank. Each GenBank sequence was used as a query against the local *rbcL* and *matK* databases containing the Welsh flora sequences using a BLASTn search with NCBI default parameters. The GenBank sequences were used as downloaded, so contained a range of different fragment sizes. A query sequence was considered as successfully matched if the top Bit-Score obtained from searching for it in the Wales database matched the name of the species in GenBank. If more than one species shared a top Bit-Score or the species scored lower, this was considered an identification failure.

### Discrimination at different spatial scales

Botanical Society of the British Isles records were obtained for each 10×10 km square of Wales and for the vice-counties of Cardiganshire [48], Caernarvonshire and Anglesey at the 2×2 km square level. In total this used 891,756 plant records. A species list for each square was generated and the presence of barcode gaps (using multiple alignments) between the species was assessed using a script written in Python. Levels of discrimination were visualised using heatmap.2 from gplots in R.

## Supporting Information

Table S1 *rbcL* primers used to amplify the native and archaeophyte flowering plants and conifers of Wales.
(DOCX)

Table S2 *matK* primers used to amplify the native and archaeophyte flowering plants and conifers of Wales.
(DOCX)

Table S3 *rbcL* and *matK* sequences, corresponding to species found in the Welsh flora, downloaded from GenBank (n = 2726). Each sequence was queried against the Welsh flora databases for *rbcL* and *matK* using a BLASTn search. A correct match was determined by the top Bit-Score matching the name of that species in GenBank. GenBank accessions in bold typeface matched our DNA barcodes.
(DOCX)

Dataset S1 Specimen information with BOLD and GenBank accessions.
(XLSX)

Dataset S2 Sequence quality scores.
(XLSX)

Dataset S3 Discrimination for each species using BLAST, Neighbour-Joining trees and barcode gap analysis with pairwise and multiple alignments.
(XLSX)
