# Supplementary material for: DNA Barcoding the Native Flowering Plants and Conifers of Wales
Source: PLoS One. 2012 Jun 6;7(6):e37945. doi: 10.1371/journal.pone.0037945 (PMC3368937; doi:10.1371/journal.pone.0037945)
Supplement: Table S1 — rbcL primers used to amplify the native and archaeophyte flowering plants and conifers of Wales. (DOCX) [file pone.0037945.s001.docx]

Table S1*. rbcL* primers used to amplify the native and archaeophyte flowering plants and conifers of Wales.

* denotes primers designed for use in this study.

| **Primer** | **F/R** | **Sequence 5' - 3'** | **Reference** |
| --- | --- | --- | --- |
| rbcLa-F | F | ATGTCACCACAAACAGAGACTAAAGC | [1] |
| rbcLr590 | R | AGTCCACCGCGTAGACATTCAT | * |
| rbcLr506 | R | AGGGGACGACCATACTTGTTCA | * |
| rbcLa-rev | R | GTAAAATCAAGTCCACCRCG | [2] |
| rbcLajf634R | R | GAAACGGTCTCTCCAACGCAT | [3] |
| rbcL724R | R | TCGCATGTACCTGCAGTAGC | [4] |

1. Kress WJ, Erickson DL (2007) A Two-Locus Global DNA Barcode for Land Plants: The Coding *rbcL* Gene Complements the Non-Coding trnH-psbA Spacer Region. PLoS one 2: e508.

2. Kress WJ, Erickson DL, Andrew Jones F, Swenson NG, Perez R, et al. (2009) Plant DNA barcodes and a community phylogeny of a tropical forest dynamics plot in Panama. Proceedings of the National Academy of Sciences of the United States of America 106: 18621-18626.

3. Fazekas AJ, Burgess KS, Kesanakurti PR, Graham SW, Newmaster SG, et al. (2008) Multiple Multilocus DNA Barcodes from the Plastid Genome Discriminate Plant Species Equally Well. PLoS one 3: e2802.

4. Fay MF, Swensen SM, Chase MW (1997) Taxonomic affinities of *Medusagyne oppositifolia* (Medusagynaceae). Kew Bulletin 52: 111-120.
