# Supplementary material for: DNA Barcoding the Native Flowering Plants and Conifers of Wales
Source: PLoS One. 2012 Jun 6;7(6):e37945. doi: 10.1371/journal.pone.0037945 (PMC3368937; doi:10.1371/journal.pone.0037945)
Supplement: Table S2 — matK primers used to amplify the native and archaeophyte flowering plants and conifers of Wales. (DOCX) [file pone.0037945.s002.docx]

Table S2. *matK* primers used to amplify the native and archaeophyte flowering plants and conifers of Wales.

* denotes primers designed for use in this study.

| **Primer** | **F/R** | **Sequence 5' - 3'** | **Reference** |
| --- | --- | --- | --- |
| matK2.1a | F | ATCCATCTGGAAATCTTAGTTC | [1] |
| matK2.1F | F | CCTATCCATCTGGAAATCTTAG | [1] |
| matK_1R_kim | F | ACCCAGTCCATCTGGAAATCTTGGTCC | K. J. Kim, unpub. |
| MatK_390f | F | CGATCTATTCATTCAATATTTC | [2] |
| MatK_Xf | F | TAATTTACGATCAATTCATTC | [1] |
| F389 | F | GGAAATCCATTCTGGCTTCAAAAGG | * |
| F461asterales | F | TAGATATACTAATACCTTACCCAGC | * |
| F1010asterales | F | ATTCCCTTGACTTTCTGGGTTATCG | * |
| F2brassicales | F | CATTTCCCTAAGATTAGGATCCTCT | * |
| F2caryophyllales | F | TGCCCATCTAGAAATCTTGGTTCAA | * |
| F428fabales | F | CTTATAAAAATTTGCGATCAATTCATTC | * |
| F2lamiales | F | CCCCAATTTGCGATCAATTCATTCA | * |
| F318poales | F | TTCTGAGTTTTATTCTCAGATT | * |
| F875poales | F | TTATGGATCCTCTTATGCATTATGT | * |
| F2ranunculales | F | AAAACCTCCGTTGTTGGATACAAGA | * |
| F458rosales | F | CATTTAAATTATGCATCAGATGTACTA | * |
| MatK-3FKIM-r | R | CGTACAGTACTTTTGTGTTTACGAG | K.J. Kim, unpub |
| MatK_1326r | R | TCTAGCACACGAAAGTCGAAGT | [2] |
| MatK_5r | R | GTTCTAGCACAAGAAAGTCG | [1] |
| matK3.2 | R | CTTCCTCTGTAAAGAATTC | [1] |
| R692 | R | TACAAAATTGCGCTTTAGCCAATGATC | * |
| R1498asterales | R | CATTATTGGCCAAATCATTGATACA | * |
| R978asterales | R | CAGAAAGTCAAGGGAATGATTGGAT | * |
| R2brassicales | R | CCAAATACCAAATTCGCACTCGATA | * |
| R2caryophyllales | R | TGTGTTTACGAGCCAAAGTTCTAGC | * |
| R1438fabales | R | CTCTTCTTCCTCTGTAAAGAATTCT | * |
| R2lamiales | R | CAGTACTTTTGTGTTTCCGAGCCAA | * |
| R1460poales | R | AGGGTTGTTTTGGTGAACATCAAAG | * |
| R854poales | R | ATCGAACATAATGCATAAGAGGATC | * |
| R1020ranunculales | R | ATGAAGCCTATCCGGCTGAGACCAA | * |
| R2ranunculales | R | CCCCAGAAAGAACCTGTTCTTCCTC | * |
| R1414rosales | R | TCTGTTCTTCTTCCGTAAAGAATTC | * |

1. Ford CS, Ayres KL, Toomey N, Haider N, Stahl JVA, et al. (2009) Selection of candidate coding DNA barcoding regions for use on land plants. Botanical Journal of the Linnean Society 159: 1-11.

2. Cuenoud P, Savolainen V, Chatrou LW, Powell M, Grayer RJ, et al. (2002) Molecular phylogenetics of Caryophyllales based on nuclear 18S rDNA and plastid *rbcL*, *atpB*, and *matK* DNA sequences. American Journal of Botany 89: 132-144.
