# Supplementary material for: DNA Barcoding the Native Flowering Plants and Conifers of Wales
Source: PLoS One. 2012 Jun 6;7(6):e37945. doi: 10.1371/journal.pone.0037945 (PMC3368937; doi:10.1371/journal.pone.0037945)
Supplement: Table S3 — rbcL and matK sequences, corresponding to species found in the Welsh flora, downloaded from GenBank (n = 2726). Each sequence was queried against the Welsh flora databases for rbcL and matK using a BLASTn search. A correct match was determined by the top Bit-Score matching the name of that species in GenBank. GenBank accessions in bold typeface matched our DNA barcodes. (DOCX) [file pone.0037945.s003.docx]

Table S3. *rbcL* and *matK* sequences, corresponding to species found in the Welsh flora, downloaded from GenBank (n = 2726). Each sequence was queried against the Welsh flora databases for *rbcL* and *matK* using a BLASTn search. A correct match was determined by the top Bit-Score matching the name of that species in GenBank. GenBank accessions in bold typeface matched our DNA barcodes.

| **Order** | **Family** | **Species** | ***rbcL* GenBank Accession** | ***matK* GenBank Accession** |
| --- | --- | --- | --- | --- |
| Alismatales | Alismataceae | *Alisma lanceolatum* | HM849753.1 |  |
| Alismatales | Alismataceae | *Alisma plantago* | L08759.1 | **AF542573.1** |
| Alismatales | Alismataceae | *Baldellia ranunculoides* | **HM849805.1** **U80677.1** **DQ859163.1** | HM850587.1 |
| Alismatales | Alismataceae | *Luronium natans* | **U80680.1** |  |
| Alismatales | Alismataceae | *Sagittaria sagittifolia* | **GU344672.1** |  |
| Alismatales | Araceae | *Arum italicum* | HM849794.1 EU193237.1 EU193236.1 GU067578.1 | HM850480.1 EU193628.1 EU193627.1 EU886517.1 GU067603.1 FN668801.1 |
| Alismatales | Araceae | *Arum maculatum* | EU193250.1 GU067582.1 DQ005607.1 | EU193641.1 EU886506.1 GU067608.1 FN668802.1 FJ395376.1 |
| Alismatales | Araceae | *Lemna gibba* | **GU454417.1** **GU454416.1** **GU454415.1** **GU454414.1** **GU454413.1** **GU454412.1** **AY034235.1** |  |
| Alismatales | Araceae | *Lemna minor* | HM850112.1 **GU454424.1** **GU454423.1** **GU454422.1** **GU454421.1** **GU454420.1** **GU454419.1** **AY034234.1** GQ436374.1 AM905730.1 | **HM850477.1** **GU454162.1** **GU454161.1** **GU454160.1** **GU454159.1** **GU454158.1** **GU454157.1** **AY034196.1** **AM920552.1** |
| Alismatales | Araceae | *Lemna trisulca* | **GU454431.1** **GU454430.1** **GU454429.1** **AY034237.1** | **GU454169.1** **GU454168.1** **GU454167.1** **AY034199.1** |
| Alismatales | Araceae | *Spirodela polyrhiza* | **GU454401.1** **GU454400.1** **GU454399.1** **GU454398.1** **GU454397.1** **GU454396.1** **GU454395.1** **GU454394.1** **AY034222.1** **AM905731.1** | **GU454139.1** **GU454138.1** **GU454137.1** **GU454136.1** **GU454135.1** **GU454134.1** **GU454133.1** **GU454132.1** **AY034184.1** **AM920553.1** |
| Alismatales | Araceae | *Wolffia arrhiza* | **GU454461.1** **AY034254.1** |  |
| Alismatales | Butomaceae | *Butomus umbellatus* | **U80685.1** **AY149345.1** | **AY952416.1** **DQ401367.1** **AY870364.1** |
| Alismatales | Hydrocharitaceae | *Hydrocharis morsus* | **U80701.1** |  |
| Alismatales | Juncaginaceae | *Triglochin maritimum* | **U80714.1** **GQ452333.1** **AB088811.1** | AF542566.1 GQ452339.1 AB088782.1 AM920647.1 |
| Alismatales | Juncaginaceae | *Triglochin palustris* | **GQ452334.1** **DQ859176.1** | **GQ452340.1** |
| Alismatales | Potamogetonaceae | *Groenlandia densa* | **U80720.1** **AB196954.1** |  |
| Alismatales | Potamogetonaceae | *Potamogeton alpinus* | AB196845.1 |  |
| Alismatales | Potamogetonaceae | *Potamogeton berchtoldii* | FJ968699.1 FJ956852.1 |  |
| Alismatales | Potamogetonaceae | *Potamogeton coloratus* | GU344673.1 |  |
| Alismatales | Potamogetonaceae | *Potamogeton compressus* | AB196846.1 |  |
| Alismatales | Potamogetonaceae | *Potamogeton crispus* | FJ956836.1 U80722.1 AB196847.1 |  |
| Alismatales | Potamogetonaceae | *Potamogeton filiformis* | FJ956863.1 FJ956862.1 FJ956861.1 |  |
| Alismatales | Potamogetonaceae | *Potamogeton gramineus* | FJ968691.1 FJ968690.1 FJ956828.1 **U80723.1** **AB196943.1** |  |
| Alismatales | Potamogetonaceae | *Potamogeton natans* | FJ956833.1 FJ956832.1 FJ956831.1 DQ859174.1 AB196946.1 |  |
| Alismatales | Potamogetonaceae | *Potamogeton obtusifolius* | FJ956854.1 AB196947.1 |  |
| Alismatales | Potamogetonaceae | *Potamogeton pectinatus* | **U80727.1** **FJ956866.1** **FJ956865.1** **FJ956864.1** AB196953.1 |  |
| Alismatales | Potamogetonaceae | *Potamogeton perfoliatus* | FJ968700.1 **FJ968683.1** **FJ956826.1** **FJ956825.1** **AY952437.1** **U80724.1** **EU741052.1** **EU741051.1** **AB196951.1** | **AY952425.1** |
| Alismatales | Potamogetonaceae | *Potamogeton polygonifolius* | HM850282.1 | **HM851048.1** |
| Alismatales | Potamogetonaceae | *Potamogeton praelongus* | FJ968702.1 FJ968701.1 FJ968683.1 FJ956827.1 AB196952.1 |  |
| Alismatales | Potamogetonaceae | *Potamogeton pusillus* | HM850283.1 FJ968699.1 FJ968698.1 FJ956846.1 FJ956845.1 AB250148.1 AB196950.1 | HM851049.1 |
| Alismatales | Potamogetonaceae | *Zannichellia palustris* | **U03725.1** **AB196955.1** |  |
| Alismatales | Ruppiaceae | *Ruppia maritima* | **HM850324.1** **U03729.1** | **AB507928.1** **AB507927.1** **AB507926.1** **AB507925.1** **AB507924.1** **AB507923.1** **AB507922.1** **AB507921.1** **AB507920.1** **AB507919.1** **AB507918.1** **AB507917.1** **AB507916.1** **AB507915.1** **AB507914.1** **AB507913.1** **AB507912.1** **AB507911.1** **AB507910.1** **AB507909.1** |
| Alismatales | Zosteraceae | *Zostera marina* | **U80734.1** U03724.1 **AB125349.1** **AB125348.1** |  |
| Apiales | Apiaceae | *Aegopodium podagraria* | U50220.1 | U58542.1 |
| Apiales | Apiaceae | *Angelica sylvestris* | **DQ133798.1** | **DQ133783.1** |
| Apiales | Apiaceae | *Anthriscus sylvestris* | **HE574581.1** **FJ395576.1** | **FJ395382.1** **U58547.1** |
| Apiales | Apiaceae | *Apium graveolens* | **HM849783.1** **L01885.2** AF479575.1 | **HM850719.1** **U58548.1** **AJ429370.1** |
| Apiales | Apiaceae | *Apium nodiflorum* | HM850050.1 |  |
| Apiales | Apiaceae | *Berula erecta* | AM234813.1 |  |
| Apiales | Apiaceae | *Chaerophyllum temulum* | **FJ395594.1** | **FJ395423.1** |
| Apiales | Apiaceae | *Conium maculatum* | **HM849908.1** L11167.1 | **HM850724.1** |
| Apiales | Apiaceae | *Crithmum maritimum* | HM849924.1 | **HM850727.1** **U58558.1** |
| Apiales | Apiaceae | *Daucus carota* | **HM849948.1** **HQ619726.1** GQ220324.1 **AF207689.1** **FJ395569.1** | **HM850728.1** **HQ619790.1** **GQ434234.1** **FJ395374.1** **U58559.1** |
| Apiales | Apiaceae | *Foeniculum vulgare* | **HM850008.1** **GQ120445.1** | **HM850711.1** **GQ434233.1** **U58563.1** **EU531667.1** |
| Apiales | Apiaceae | *Heracleum sphondylium* | AY395540.1 FJ395586.1 | **FJ395398.1** |
| Apiales | Apiaceae | *Pastinaca sativa* |  | **U58573.1** |
| Apiales | Apiaceae | *Petroselinum crispum* | **HM850248.1** **AY188433.1** | HM850712.1 AY188405.1 U58575.1 |
| Apiales | Apiaceae | *Pimpinella saxifraga* | **FR865128.1** **FJ395558.1** **U50229.1** | **FR865050.1** **FJ395362.1** **U58576.1** |
| Apiales | Apiaceae | *Sanicula europaea* | **DQ133820.1** | **FJ395370.1** |
| Apiales | Apiaceae | *Torilis arvensis* | **HM850403.1** **AM234827.1** |  |
| Apiales | Apiaceae | *Torilis japonica* | **FJ395562.1** |  |
| Apiales | Apiaceae | *Torilis nodosa* | HM850404.1 | HM850715.1 |
| Apiales | Araliaceae | *Hedera helix* | **HQ619729.1** **L01924.2** **FN689376.1** **FJ395559.1** | **HQ619793.1** **FN668806.1** **FN668805.1** **FJ395363.1** **U58612.1** **AM503815.2** **AJ319074.1** **AJ319072.1** **AJ319070.1** **AJ319068.1** **AJ319066.1** **AJ319075.1** **AJ319073.1** **AJ319071.1** **AJ319069.1** **AJ319067.1** |
| Apiales | Araliaceae | *Hydrocotyle vulgaris* | **HM850058.1** **DQ133813.1** | **HM850710.1** **DQ133792.1** |
| Aquifoliales | Aquifoliaceae | *Ilex aquifolium* | **FJ394589.1** **FJ395601.1** **X69741.1** | **AF542607.2** FN668798.1 **FJ395435.1** |
| Asparagales | Amaryllidaceae | *Allium ampeloprasum* | HM849754.1 | **HM850502.1** |
| Asparagales | Amaryllidaceae | *Allium vineale* | HM849756.1 | HM850504.1 |
| Asparagales | Asparagaceae | *Asparagus officinalis* | HM849798.1 L05028.2 AY149374.1 |  |
| Asparagales | Asparagaceae | *Convallaria majalis* | **HM640443.1** **EU850070.1** **GQ436294.1** **AB089627.1** **D28334.1** | **HM640557.1** **FN668803.1** **GQ434046.1** **AB029771.1** |
| Asparagales | Asparagaceae | *Hyacinthoides nonscripta* | **FJ395578.1** |  |
| Asparagales | Asparagaceae | *Polygonatum multiflorum* |  | **EF133696.1** |
| Asparagales | Asparagaceae | *Polygonatum odoratum* | **HM640459.1** | HM640573.1 GQ434068.1 **AB017316.1** |
| Asparagales | Asparagaceae | *Ruscus aculeatus* | **HM640440.1** **HM850325.1** **AB088822.1** **Z77274.1** | **HM640554.1** **HM850496.1** **FN668804.1** **AB029801.1** |
| Asparagales | Iridaceae | *Iris foetidissima* | **HM850074.1** | **HM850489.1** **FJ197278.1** |
| Asparagales | Orchidaceae | *Coeloglossum viride* |  | **EF079300.1** **AM883576.1** |
| Asparagales | Orchidaceae | *Dactylorhiza fuchsii* |  | EU214325.1 AM883561.1 |
| Asparagales | Orchidaceae | *Dactylorhiza incarnata* |  | **AM883570.1** **AM883564.1** **AM883563.1** **AM883559.1** |
| Asparagales | Orchidaceae | *Dactylorhiza maculata* | GQ248587.1 | **GQ248115.1** **EU214326.1** **EF612529.1** **AY708007.1** **AM883568.1** **AM883566.1** |
| Asparagales | Orchidaceae | *Epipactis helleborine* | Z73707.1 | EU490692.1 AF263659.1 |
| Asparagales | Orchidaceae | *Epipactis leptochila* | FJ454879.1 |  |
| Asparagales | Orchidaceae | *Epipactis palustris* | FJ454882.1 |  |
| Asparagales | Orchidaceae | *Gymnadenia conopsea* |  | EF612530.1 AY708008.1 **AM883569.1** |
| Asparagales | Orchidaceae | *Liparis loeselii* |  | **AY907157.1** |
| Asparagales | Orchidaceae | *Neottia nidus* | AY368364.1 |  |
| Asparagales | Orchidaceae | *Ophrys apifera* | **AF074202.1** **AJ542396.1** | **AJ543953.1** **AJ310049.1** |
| Asparagales | Orchidaceae | *Platanthera chlorantha* |  | EF612531.1 **DQ522103.1** AY708009.1 |
| Asparagales | Orchidaceae | *Spiranthes spiralis* | **FJ571355.1** **AJ542434.1** | **AJ543918.1** |
| Asterales | Asteraceae | *Achillea millefolium* | HE574579.1 HM849740.1 EU384938.1 L13641.1 | **HM850607.1** **EU385315.1** **FJ395393.1** |
| Asterales | Asteraceae | *Achillea ptarmica* | HE574580.1 |  |
| Asterales | Asteraceae | *Antennaria dioica* | **HE574602.1** | **HM445620.1** |
| Asterales | Asteraceae | *Anthemis arvensis* | HM849778.1 | HM850610.1 |
| Asterales | Asteraceae | *Anthemis cotula* | **HM849779.1** | **HM850611.1** |
| Asterales | Asteraceae | *Arctium lappa* | GU724233.1 GQ436460.1 GQ436459.1 AB530978.1 | HM989770.1 HM989769.1 GQ434116.1 AY013520.1 |
| Asterales | Asteraceae | *Arctium minus* | HM849788.1 | HM850616.1 AY013521.1 FJ395403.1 |
| Asterales | Asteraceae | *Bellis perennis* | **HM849813.1** **AY395530.1** **AB530952.1** | **HM850613.1** |
| Asterales | Asteraceae | *Bidens tripartita* | HE574604.1 HE574582.1 | AY551475.1 |
| Asterales | Asteraceae | *Carduus tenuiflorus* | HM849849.1 | HM850617.1 |
| Asterales | Asteraceae | *Carlina vulgaris* |  | **AY013533.1** |
| Asterales | Asteraceae | *Centaurea cyanus* | **HE574606.1** AB530955.1 |  |
| Asterales | Asteraceae | *Centaurea nigra* |  | **FJ395405.1** |
| Asterales | Asteraceae | *Chamaemelum nobile* | **HM849885.1** | HM850619.1 AF456775.1 |
| Asterales | Asteraceae | *Chrysanthemum segetum* | **HM849893.1** |  |
| Asterales | Asteraceae | *Cichorium intybus* | **HM849895.1** **HQ619731.1** **L13652.1** **AB530980.1** | **HM850620.1** **HQ619795.1** GU817441.1 **AJ633132.1** **AJ633134.1** **AJ633133.1** **AJ633131.1** |
| Asterales | Asteraceae | *Cirsium arvense* | HM849897.1 AY395534.1 | **HM850621.1** **FJ395428.1** |
| Asterales | Asteraceae | *Cirsium palustre* | HM849898.1 | HM850622.1 AY013536.1 |
| Asterales | Asteraceae | *Cirsium vulgare* | HM849899.1 | HM850623.1 |
| Asterales | Asteraceae | *Crepis capillaris* | HM849923.1 | HM850628.1 **FJ395373.1** AJ633142.1 |
| Asterales | Asteraceae | *Crepis mollis* |  | **DQ451748.1** **EU363538.1** |
| Asterales | Asteraceae | *Eupatorium cannabinum* |  | **AB217692.1** **AB217691.1** |
| Asterales | Asteraceae | *Filago minima* |  | HM850632.1 |
| Asterales | Asteraceae | *Gnaphalium uliginosum* |  | **HM445642.1** |
| Asterales | Asteraceae | *Hypochaeris glabra* | HM850068.1 | HM850665.1 AJ633232.1 |
| Asterales | Asteraceae | *Hypochaeris radicata* | HM850069.1 AY395542.1 FJ395583.1 | **HM850666.1** **FJ395390.1** **AJ633231.1** |
| Asterales | Asteraceae | *Inula crithmoides* | **AY545884.1** |  |
| Asterales | Asteraceae | *Inula helenium* |  | **AF151473.1** |
| Asterales | Asteraceae | *Lactuca serriola* | HM850098.1 EU676929.1 EU676928.1 | HM850642.1 GU109315.1 EU749322.1 EU749321.1 AJ633237.1 |
| Asterales | Asteraceae | *Lactuca virosa* |  | AJ633239.1 |
| Asterales | Asteraceae | *Lapsana communis* | **HM850099.1** | **HM850644.1** **FJ395399.1** **AJ633138.1** **AJ633137.1** |
| Asterales | Asteraceae | *Leontodon autumnalis* | HE574613.1 | **DQ451694.1** AJ633222.1 AJ633220.1 |
| Asterales | Asteraceae | *Leontodon hispidus* | FR865147.1 AY395545.1 | **FR865059.1** **DQ451708.1** **DQ451707.1** **DQ451706.1** **AJ633218.1** |
| Asterales | Asteraceae | *Leontodon saxatilis* | **HM850115.1** | **HM850649.1** **DQ451726.1** **DQ451725.1** **AJ633223.1** |
| Asterales | Asteraceae | *Leucanthemum vulgare* | **HE574589.1** **HE574588.1** **HM850122.1** **AY395546.1** **AB530957.1** | **HM850667.1** **FJ395389.1** |
| Asterales | Asteraceae | *Mycelis muralis* |  | **AJ633236.1** |
| Asterales | Asteraceae | *Picris echioides* | **HM850259.1** | **HM850657.1** **DQ451730.1** **AJ633230.1** |
| Asterales | Asteraceae | *Picris hieracioides* |  | **AJ633229.1** |
| Asterales | Asteraceae | *Scorzonera humilis* |  | AJ633268.1 |
| Asterales | Asteraceae | *Senecio aquaticus* | HM850080.1 AY395561.1 | AF460014.1 |
| Asterales | Asteraceae | *Senecio erucifolius* | **FJ395566.1** |  |
| Asterales | Asteraceae | *Senecio jacobaea* | GU817769.1 | AF460007.1 AY156991.1 AY156990.1 AY156989.1 AY156988.1 AY156987.1 AY156986.1 AY156985.1 AY156984.1 AY156983.1 AY156982.1 AY156981.1 AY156980.1 FJ395360.1 |
| Asterales | Asteraceae | *Senecio sylvaticus* | HM850346.1 | AF459998.1 |
| Asterales | Asteraceae | *Senecio vulgaris* | HM850347.1 HQ619732.1 FJ395580.1 | HM850674.1 HQ619796.1 AF459995.1 FJ395385.1 DQ208169.1 AF151509.1 |
| Asterales | Asteraceae | *Silybum marianum* | **HM850356.1** |  |
| Asterales | Asteraceae | *Solidago virgaurea* | **HE574593.1** |  |
| Asterales | Asteraceae | *Sonchus arvensis* |  | **DQ840453.1** **DQ508002.1** **DQ508001.1** |
| Asterales | Asteraceae | *Sonchus asper* | HM850372.1 EU677034.1 EU677033.1 EU677032.1 | HM850661.1 EU749426.1 EU749425.1 EU749424.1 DQ840454.1 DQ840426.1 DQ508004.1 DQ508003.1 DQ022963.1 FJ395412.1 |
| Asterales | Asteraceae | *Sonchus oleraceus* | HM850373.1 EU385018.1 EU677036.1 EU677035.1 | HM850662.1 EU385397.1 EU749427.1 DQ840449.1 DQ840448.1 DQ840447.1 AJ633279.1 |
| Asterales | Asteraceae | *Tanacetum parthenium* | **HM850390.1** |  |
| Asterales | Asteraceae | *Tragopogon pratensis* | **AY395563.1** | **FJ395443.1** |
| Asterales | Asteraceae | *Tussilago farfara* |  | **EF537951.1** |
| Asterales | Campanulaceae | *Campanula latifolia* | EU713378.1 FJ587260.1 EF141027.1 | **EU713271.1** |
| Asterales | Campanulaceae | *Campanula rapunculus* | **FJ587272.1** |  |
| Asterales | Campanulaceae | *Campanula rotundifolia* | HE574583.1 **FR865133.1** **EU713443.1** **EU713442.1** **FJ587273.1** | **FR865054.1** **EU713336.1** **EU713335.1** |
| Asterales | Campanulaceae | *Campanula trachelium* | FJ587285.1 DQ356118.1 |  |
| Asterales | Campanulaceae | *Jasione montana* | **EU643731.1** **EU713354.1** **DQ356120.1** | **EU713247.1** |
| Asterales | Campanulaceae | *Lobelia dortmanna* | **DQ356162.1** |  |
| Asterales | Campanulaceae | *Wahlenbergia hederacea* | **EU643708.1** **EU713400.1** | **EU713293.1** |
| Asterales | Menyanthaceae | *Menyanthes trifoliata* | **EF173094.1** **EF173093.1** **L14006.2** | **EF173062.1** **AJ429386.1** |
| Boraginales | Boraginaceae | *Cynoglossum officinale* | **EU599840.1** | **EU599664.1** |
| Boraginales | Boraginaceae | *Echium vulgare* |  | **FJ827257.1** **EU919617.1** **FJ789898.1** **AY092893.1** |
| Boraginales | Boraginaceae | *Lithospermum arvense* | **EU599852.1** **HM849830.1** | **EU599676.1** **HM850865.1** |
| Boraginales | Boraginaceae | *Lithospermum officinale* | **EU599855.1** | **EU599679.1** **FJ827267.1** EU919619.1 **FJ789911.1** |
| Boraginales | Boraginaceae | *Myosotis arvensis* | EU599847.1 HM850186.1 | EU599671.1 HM850871.1 **AY092861.1** |
| Boraginales | Boraginaceae | *Myosotis discolor* | HM850187.1 AY395552.1 | **HM850872.1** **AY092870.1** |
| Boraginales | Boraginaceae | *Myosotis laxa* | HE574615.1 | AY092865.1 |
| Boraginales | Boraginaceae | *Myosotis ramosissima* | HE574633.1 |  |
| Boraginales | Boraginaceae | *Myosotis scorpioides* | GU344681.1 |  |
| Boraginales | Boraginaceae | *Myosotis secunda* |  | **HM850874.1** |
| Boraginales | Boraginaceae | *Myosotis sylvatica* | HE574616.1 | AY092887.1 **FJ395375.1** |
| Brassicales | Brassicaceae | *Alliaria petiolata* | **FJ395597.1** | **AF144363.1** |
| Brassicales | Brassicaceae | *Arabidopsis thaliana* | **HM849785.1** DQ310545.1 DQ310544.1 DQ310543.1 DQ310542.1 DQ310541.1 DQ310540.1 DQ310539.1 DQ310538.1 **AY174638.1** **AY174637.1** **AY174636.1** **AY174635.1** **AY174634.1** **AY174633.1** **AY174632.1** **AB003522.1** | **HM850734.1** HQ616528.2 **AF144378.1** **AF144377.1** **AF144376.1** **AF144375.1** **AF144374.1** **AF144373.1** **AF144372.1** **AF144371.1** **AF144370.1** **AF144348.1** **AJ971667.1** **AJ971666.1** |
| Brassicales | Brassicaceae | *Arabis hirsuta* | FR865132.1 AP009369.1 | **FR865053.1** **AF144338.1** **AP009369.1** |
| Brassicales | Brassicaceae | *Armoracia rusticana* | **AF020323.1** | **FN597648.1** |
| Brassicales | Brassicaceae | *Barbarea vulgaris* | **HE574603.1** | **EU371818.1** **AF144330.1** |
| Brassicales | Brassicaceae | *Brassica nigra* | HM849822.1 | **HM850744.1** **AB354272.1** |
| Brassicales | Brassicaceae | *Brassica oleracea* | **HM849824.1** **HQ619736.1** **GQ184380.1** **GQ184379.1** **GQ184378.1** **GQ184377.1** **GQ184376.1** **GQ184375.1** **M88342.1** | HM850745.1 HQ619800.1 AY541622.1 AY541621.1 AY541620.1 AY541619.1 AY541618.1 AY541617.1 AY541616.1 AY541615.1 AY541614.1 AY541613.1 AY541612.1 AY541611.1 **AB354271.1** |
| Brassicales | Brassicaceae | *Brassica rapa* | **GQ184371.1** GQ184370.1 **GQ184369.1** GQ184367.1 **GQ184366.1** **GQ184365.1** **GQ184364.1** AF479573.1 | **AY541619.1** **AB354276.1** |
| Brassicales | Brassicaceae | *Cakile maritima* | **AY167981.1** | **GQ424577.1** |
| Brassicales | Brassicaceae | *Capsella bursa* | **HQ619738.1** **FN594844.1** **DQ310539.1** **FJ395587.1** **AP009371.1** **D88904.1** | **HQ619802.1** **AP009371.1** |
| Brassicales | Brassicaceae | *Cardamine amara* |  | AF144337.1 |
| Brassicales | Brassicaceae | *Cardamine flexuosa* | D88905.1 | **GQ424604.1** |
| Brassicales | Brassicaceae | *Cardamine hirsuta* | **HM849847.1** **HQ619739.1** | **HM850748.1** **HQ619803.1** **HQ616528.2** |
| Brassicales | Brassicaceae | *Cardamine pratensis* | **HM849848.1** | **HM850749.1** |
| Brassicales | Brassicaceae | *Cochlearia danica* |  | AF174531.1 |
| Brassicales | Brassicaceae | *Cochlearia pyrenaica* |  | AF144357.1 |
| Brassicales | Brassicaceae | *Crambe maritima* |  | **GQ424580.1** |
| Brassicales | Brassicaceae | *Descurainia sophia* |  | **GQ424581.1** |
| Brassicales | Brassicaceae | *Erophila verna* | HQ619740.1 | HQ619804.1 |
| Brassicales | Brassicaceae | *Erysimum cheiranthoides* | DQ006076.1 |  |
| Brassicales | Brassicaceae | *Lepidium campestre* |  | **AF144359.1** |
| Brassicales | Brassicaceae | *Nasturtium officinale* | HM850197.1 AF020325.1 AP009376.1 | HM850754.1 AY483225.1 AP009376.1 |
| Brassicales | Brassicaceae | *Raphanus raphanistrum* | HM850299.1 **EF590570.1** | **HM850755.1** **GQ248192.1** **EF590417.1** **AB354269.1** **AB354268.1** **AB354267.1** **AB354266.1** **AB354265.1** **AB354264.1** **AB354263.1** AB182355.1 AB182350.1 |
| Brassicales | Brassicaceae | *Rorippa amphibia* | AF020327.1 | AF174530.1 |
| Brassicales | Brassicaceae | *Rorippa palustris* |  | AF144355.1 |
| Brassicales | Brassicaceae | *Rorippa sylvestris* | AF020328.1 |  |
| Brassicales | Brassicaceae | *Sinapis alba* | HM849823.1 X73284.1 | **HM850738.1** **AB354277.1** |
| Brassicales | Brassicaceae | *Sisymbrium officinale* | **HM850358.1** | **HM850740.1** **FJ395359.1** |
| Brassicales | Brassicaceae | *Teesdalia nudicaulis* |  | **GQ424601.1** |
| Brassicales | Brassicaceae | *Thlaspi arvense* | **FN594829.1** | **HM850750.1** **GQ424602.1** **AF144360.1** |
| Brassicales | Brassicaceae | *Thlaspi caerulescens* | **FN594826.1** |  |
| Brassicales | Resedaceae | *Reseda lutea* | AY483273.1 | **AY483241.1** **FM179932.1** |
| Brassicales | Resedaceae | *Reseda luteola* | **HM850302.1** **FJ212219.1** | **HM850757.1** **FJ212206.1** |
| Caryophyllales | Amaranthaceae | *Atriplex patula* | HM849801.1 | AY042550.1 HM850759.1 FJ395372.1 |
| Caryophyllales | Amaranthaceae | *Atriplex prostrata* | HM849802.1 | HM850760.1 |
| Caryophyllales | Amaranthaceae | *Beta vulgaris* | **AY270065.1** **HM849814.1** GQ220324.1 | **HM850762.1** **AY514832.1** **DQ116790.1** |
| Caryophyllales | Amaranthaceae | *Chenopodium bonus* | **AY270079.1** | **AY042567.1** **AF204864.1** **AY514834.1** |
| Caryophyllales | Amaranthaceae | *Chenopodium murale* | HM849890.1 | HM850765.1 |
| Caryophyllales | Amaranthaceae | *Chenopodium urbicum* | **HM587596.1** |  |
| Caryophyllales | Amaranthaceae | *Salicornia dolichostachya* | AY270125.1 | DQ468650.1 |
| Caryophyllales | Amaranthaceae | *Salicornia europaea* | HM131777.1 |  |
| Caryophyllales | Amaranthaceae | *Salsola kali* | **AY270129.1** **HM850332.1** | **HM850761.1** **AY514843.1** |
| Caryophyllales | Amaranthaceae | *Sarcocornia perennis* |  | **DQ468646.1** |
| Caryophyllales | Amaranthaceae | *Suaeda maritima* | **AY270137.1** **FJ436011.1** | **DQ499413.1** **DQ468647.1** |
| Caryophyllales | Amaranthaceae | *Suaeda vera* |  | **AY042658.1** |
| Caryophyllales | Caryophyllaceae | *Agrostemma githago* |  | **AY042539.1** **FJ589503.1** **AY936315.1** |
| Caryophyllales | Caryophyllaceae | *Arenaria serpyllifolia* |  | **FJ869016.1** **AY936302.1** |
| Caryophyllales | Caryophyllaceae | *Cerastium fontanum* | HM849881.1 FJ395574.1 | **HM850786.1** FJ404829.1 **AY936296.1** |
| Caryophyllales | Caryophyllaceae | *Cerastium glomeratum* | **HM849882.1** **HQ619745.1** **M83542.1** |  |
| Caryophyllales | Caryophyllaceae | *Dianthus armeria* |  | GU441120.1 GU441119.1 FJ404832.1 |
| Caryophyllales | Caryophyllaceae | *Dianthus deltoides* | HE574625.1 | **GU441150.1** **GU441149.1** |
| Caryophyllales | Caryophyllaceae | *Honckenya peploides* |  | **AY042602.1** **FJ595523.1** |
| Caryophyllales | Caryophyllaceae | *Moehringia trinervia* |  | **AY042615.1** |
| Caryophyllales | Caryophyllaceae | *Moenchia erecta* |  | **FJ404853.1** |
| Caryophyllales | Caryophyllaceae | *Myosoton aquaticum* |  | **FJ404855.1** |
| Caryophyllales | Caryophyllaceae | *Sagina apetala* | HM850327.1 | HM850780.1 |
| Caryophyllales | Caryophyllaceae | *Sagina maritima* | HM850328.1 | **HM850781.1** |
| Caryophyllales | Caryophyllaceae | *Sagina procumbens* | HM850329.1 | **HM850782.1** |
| Caryophyllales | Caryophyllaceae | *Saponaria officinalis* | **HM850338.1** | **HM850784.1** **GU441228.1** **AY936325.1** |
| Caryophyllales | Caryophyllaceae | *Scleranthus annuus* | AY270145.1 | **FJ404869.1** |
| Caryophyllales | Caryophyllaceae | *Scleranthus perennis* |  | **FJ404871.1** AY514847.1 |
| Caryophyllales | Caryophyllaceae | *Silene acaulis* |  | FJ404872.1 **EF547235.1** |
| Caryophyllales | Caryophyllaceae | *Silene conica* | **EF418564.1** |  |
| Caryophyllales | Caryophyllaceae | *Silene dioica* | EF646928.1 EF646927.1 EF646926.1 EF646925.1 EF646924.1 EF646923.1 EF646922.1 EF646921.1 EF646920.1 EF646919.1 EF646918.1 EF646917.1 EF646916.1 EF646915.1 EF646914.1 EF418567.1 | FN821164.1 FN821163.1 FN821162.1 FN821161.1 FN821160.1 FN821159.1 FN821158.1 FN821157.1 EF646913.1 EF646912.1 **EF646911.1** **EF646910.1** **EF646909.1** EF646908.1 EF646907.1 EF646906.1 EF646905.1 EF646904.1 **EF646903.1** **EF646902.1** |
| Caryophyllales | Caryophyllaceae | *Silene flos* | **AY395549.1** |  |
| Caryophyllales | Caryophyllaceae | *Silene gallica* | **HM850354.1** **M83544.1** | **HM850773.1** **FJ589528.1** |
| Caryophyllales | Caryophyllaceae | *Silene latifolia* | DQ006081.1 EU677006.1 EU677005.1 EF647098.1 EF647097.1 EF647096.1 EF647095.1 EF647094.1 EF647093.1 EF647092.1 **EF647091.1** EF647090.1 EF647089.1 EF647088.1 EF647087.1 EF647086.1 EF647085.1 EF647084.1 EF647083.1 | FN821263.2 FN821202.1 FN821186.1 FN821185.1 FN821184.1 FN821183.1 FN821182.1 FN821181.1 FN821180.1 FN821179.1 FN821178.1 FN821177.1 FN821176.1 FN821175.1 FN821174.1 FN821173.1 FN821172.1 EU749398.1 EU749397.1 EF647048.1 |
| Caryophyllales | Caryophyllaceae | *Silene noctiflora* | **EF418556.1** | **FN821194.1** **FN821193.1** **EF547240.1** |
| Caryophyllales | Caryophyllaceae | *Silene nutans* |  | **AF542598.1** **EF547241.1** |
| Caryophyllales | Caryophyllaceae | *Silene uniflora* | HM850355.1 | **FJ589565.1** **DQ841761.1** |
| Caryophyllales | Caryophyllaceae | *Silene viscaria* | **HE574614.1** | **FJ589570.1** |
| Caryophyllales | Caryophyllaceae | *Silene vulgaris* | HE574592.1 EU677009.1 EU677008.1 EU677007.1 EF646883.1 | **EU749401.1** **EU749400.1** **EU749399.1** **FJ376828.1** **EF646878.1** **EF547245.1** DQ841762.1 DQ841761.1 **DQ841760.1** DQ841759.1 DQ841758.1 **DQ841757.1** DQ841756.1 **DQ841755.1** DQ841754.1 **DQ841753.1** |
| Caryophyllales | Caryophyllaceae | *Spergula arvensis* | HM850378.1 | **HM850775.1** **AY936310.1** |
| Caryophyllales | Caryophyllaceae | *Spergularia marina* | **HM850381.1** | **HM850777.1** **AY936309.1** |
| Caryophyllales | Caryophyllaceae | *Spergularia media* |  | FJ404874.1 |
| Caryophyllales | Caryophyllaceae | *Spergularia rubra* |  | **AY936308.1** |
| Caryophyllales | Caryophyllaceae | *Stellaria graminea* | **FJ395572.1** | **FJ395378.1** |
| Caryophyllales | Caryophyllaceae | *Stellaria holostea* | **FJ395575.1** |  |
| Caryophyllales | Caryophyllaceae | *Stellaria media* | HM850386.1 AF206823.1 M62570.1 | HM850779.1 FJ404877.1 AY936299.1 |
| Caryophyllales | Caryophyllaceae | *Stellaria nemorum* |  | **AY936298.1** |
| Caryophyllales | Droseraceae | *Drosera anglica* | **AB355692.1** **AB298091.1** **AB072517.1** |  |
| Caryophyllales | Droseraceae | *Drosera rotundifolia* | **AB355691.1** **AB298089.1** **AB072538.1** |  |
| Caryophyllales | Montiaceae | *Montia fontana* |  | **AY764119.1** **AY764118.1** |
| Caryophyllales | Plumbaginaceae | *Armeria maritima* | **HM849792.1** | **AY042548.1** **HM851064.1** **GQ901433.1** **GQ901432.1** **GQ901431.1** |
| Caryophyllales | Plumbaginaceae | *Limonium vulgare* | Y16904.1 |  |
| Caryophyllales | Polygonaceae | *Fallopia convolvulus* | HM850000.1 HM357893.1 EU676948.1 EU676947.1 FM883612.1 | **HM851071.1** **GU373526.1** **EU749341.1** **EU749340.1** |
| Caryophyllales | Polygonaceae | *Fallopia dumetorum* | HM357894.1 FM883613.1 |  |
| Caryophyllales | Polygonaceae | *Oxyria digyna* | **FJ154454.1** **EU840291.1** EU554018.1 |  |
| Caryophyllales | Polygonaceae | *Persicaria amphibia* | **AY395553.1** **EF653777.1** **EF653776.1** **FM883621.1** | **EF653725.1** **EF653724.1** |
| Caryophyllales | Polygonaceae | *Persicaria bistorta* | **EU554011.1** **FM883607.1** | **AF204859.1** |
| Caryophyllales | Polygonaceae | *Persicaria hydropiper* | **EF653780.1** **EF653779.1** HM357898.1 **EU676950.1** **EU676949.1** FM883629.1 **AB008781.1** **D86291.1** | EF653728.1 EF653727.1 HM357924.1 **EU749343.1** **EU749342.1** |
| Caryophyllales | Polygonaceae | *Persicaria lapathifolia* | **HM850245.1** **EF653781.1** HM357899.1 **HM357896.1** **FM883630.1** **EU554022.1** | **HM851076.1** **EU196941.1** **EU196940.1** **EF653729.1** HM357925.1 HM357922.1 **GQ434294.1** |
| Caryophyllales | Polygonaceae | *Persicaria maculosa* | HM850246.1 EU676953.1 EU676952.1 EU676951.1 | **HM851077.1** **EU196945.1** **EU749346.1** **EU749345.1** **EU749344.1** |
| Caryophyllales | Polygonaceae | *Persicaria minor* | FM883633.1 | EU196948.1 EU196947.1 |
| Caryophyllales | Polygonaceae | *Polygonum aviculare* | HM850273.1 EF653761.1 HM357887.1 AF297127.1 EU676946.1 EU676945.1 EU676944.1 EU676943.1 EU554012.1 | HM851078.1 EF653710.1 HM357913.1 EU749339.1 EU749338.1 EU749337.1 EU749336.1 EF438020.1 |
| Caryophyllales | Polygonaceae | *Polygonum rurivagum* | FM883642.1 |  |
| Caryophyllales | Polygonaceae | *Rumex acetosa* | **AY395559.1** **FJ395573.1** **EU554017.1** | **GQ434303.1** **FJ395379.1** |
| Caryophyllales | Polygonaceae | *Rumex acetosella* | **HM850316.1** D86290.1 | **HM851080.1** **EF438022.1** |
| Caryophyllales | Polygonaceae | *Rumex conglomeratus* | HM850319.1 | HM851083.1 |
| Caryophyllales | Polygonaceae | *Rumex crispus* | HM850320.1 EU840290.1 | HM851084.1 FJ395409.1 |
| Caryophyllales | Polygonaceae | *Rumex obtusifolius* | HM850322.1 AF297126.1 | HM851086.1 EF438023.1 |
| Caryophyllales | Polygonaceae | *Rumex pulcher* | HM850323.1 |  |
| Caryophyllales | Polygonaceae | *Rumex sanguineus* |  | FJ395432.1 |
| Celastrales | Parnassiaceae | *Parnassia palustris* | **AY935732.1** **AY935731.1** | **AY935911.1** **AY935910.1** |
| Ceratophyllales | Ceratopyllaceae | *Ceratophyllum demersum* | **HM849883.1** **M77030.1** **D89473.1** | **HM850521.1** **AY335985.1** **AY335984.1** **AF543732.1** AF465289.1 AJ966794.1 |
| Ceratophyllales | Ceratopyllaceae | *Ceratophyllum submersum* | AF197599.1 | DQ401361.1 **AY335987.1** AJ581400.1 |
| Cornales | Cornaceae | *Cornus sanguinea* | **FJ395589.1** | **DQ340472.1** **FJ395411.1** |
| Cucurbitales | Cucurbitaceae | *Bryonia dioica* | **DQ535786.1** | **DQ536641.1** |
| Dioscoreales | Dioscoreaceae | *Tamus communis* | **HM849954.1** **FR865161.1** **AF307474.1** **FJ395602.1** | **HM850486.1** **AY973843.1** **AF465303.1** **FJ395436.1** **AJ581408.1** |
| Dioscoreales | Nartheciaceae | *Narthecium ossifragum* | **AY149348.1** **AJ286560.1** |  |
| Dipsacales | Adoxaceae | *Adoxa moschatellina* | **L01883.2** **AF446930.1** **FJ395577.1** | **AF446900.1** **FJ395383.1** |
| Dipsacales | Caprifoliaceae | *Lonicera periclymenum* |  | **AF542609.1** |
| Dipsacales | Caprifoliaceae | *Sambucus ebulus* |  | **FN668834.1** |
| Dipsacales | Caprifoliaceae | *Sambucus nigra* | **HM850334.1** **FJ395598.1** AJ420867.1 | HM850979.1 FN668836.1 AY310458.1 **FJ395429.1** |
| Dipsacales | Caprifoliaceae | *Viburnum lantana* | **HQ619743.1** | **HQ619808.1** |
| Dipsacales | Caprifoliaceae | *Viburnum opulus* | **EU677071.1** **EU677070.1** | **EU749462.1** **EU749461.1** |
| Dipsacales | Dipsacaceae | *Dipsacus fullonum* |  | **FJ745400.1** |
| Dipsacales | Dipsacaceae | *Knautia arvensis* | **HE574586.1** |  |
| Dipsacales | Dipsacaceae | *Scabiosa columbaria* | **AF446948.1** | **AF446918.1** |
| Dipsacales | Dipsacaceae | *Succisa pratensis* |  | **FJ745401.1** |
| Dipsacales | Valerianaceae | *Valeriana dioica* | AY362489.1 | **AY362531.1** **AY310468.1** |
| Dipsacales | Valerianaceae | *Valeriana officinalis* | **L13934.1** **AY362490.1** | **AY362532.1** **AY310467.1** |
| Dipsacales | Valerianaceae | *Valerianella eriocarpa* |  | AY310466.1 |
| Dipsacales | Valerianaceae | *Valerianella locusta* | AF446954.1 Y10707.1 | AY310465.1 AF446922.1 |
| Ericales | Balsaminaceae | *Impatiens nolitangere* | **AB043516.1** | **AF542608.2** |
| Ericales | Ericaceae | *Andromeda polifolia* | **HE574624.1** **AF419812.1** **AF124572.1** | **AF124569.1** |
| Ericales | Ericaceae | *Calluna vulgaris* | **HM849840.1** **L12601.2** | **HM850861.1** **U61326.1** |
| Ericales | Ericaceae | *Empetrum nigrum* | **AF421091.1** **AF419822.1** | **HQ115642.1** **HQ115639.1** **GU176670.1** **AF519558.1** |
| Ericales | Ericaceae | *Erica tetralix* | **AF419825.1** | **U61340.1** |
| Ericales | Ericaceae | *Orthilia secunda* | **AF419838.1** | AF440422.1 |
| Ericales | Ericaceae | *Pyrola rotundifolia* | L12622.2 | U61328.1 |
| Ericales | Ericaceae | *Vaccinium myrtillus* | **AY145447.1** **AF419836.1** | **AF382810.1** |
| Ericales | Ericaceae | *Vaccinium vitis* | **AF419837.1** | **AF382819.1** |
| Ericales | Primulaceae | *Anagallis arvensis* | **HM849770.1** **M88343.1** | **HM850730.1** |
| Ericales | Primulaceae | *Anagallis minima* | **HM849772.1** |  |
| Ericales | Primulaceae | *Anagallis tenella* | **HM849773.1** | **HM850732.1** **AJ581446.1** |
| Ericales | Primulaceae | *Glaux maritima* | **AF213821.1** |  |
| Ericales | Primulaceae | *Hottonia palustris* | **AF395002.1** | **AY647534.1** |
| Ericales | Primulaceae | *Lysimachia nummularia* | **AY395550.1** |  |
| Ericales | Primulaceae | *Lysimachia vulgaris* | **AF421095.1** |  |
| Ericales | Primulaceae | *Primula veris* | **AF394986.1** **AF394982.1** **AF394981.1** **AF394976.1** | **AY647530.1** **AJ429293.1** |
| Ericales | Primulaceae | *Primula vulgaris* | **HQ619773.1** AF394986.1 FJ395567.1 | **HQ619829.1** **DQ378362.1** |
| Ericales | Primulaceae | *Samolus valerandi* | **HM850335.1** **U96659.1** | **HM850729.1** |
| Fabales | Fabaceae | *Anthyllis vulneraria* |  | **AF543845.1** |
| Fabales | Fabaceae | *Cytisus scoparius* | **HM849943.1** **Z70086.1** | **AY386902.1** **HM851131.1** |
| Fabales | Fabaceae | *Genista tinctoria* | **Z70099.1** |  |
| Fabales | Fabaceae | *Hippocrepis comosa* | FR865156.1 |  |
| Fabales | Fabaceae | *Lathyrus japonicus* | **HM850107.1** | **HM851119.1** **HM026395.1** **HM026394.1** |
| Fabales | Fabaceae | *Lathyrus palustris* |  | **HM026396.1** |
| Fabales | Fabaceae | *Lathyrus pratensis* | **AY395544.1** |  |
| Fabales | Fabaceae | *Lotus corniculatus* | HM850139.1 U74213.1 | HM851124.1 HM049505.1 HM049504.1 |
| Fabales | Fabaceae | *Lotus pedunculatus* | HM850142.1 | HM851127.1 |
| Fabales | Fabaceae | *Medicago arabica* | **HM850163.1** | **HM851135.1** **HM159554.1** |
| Fabales | Fabaceae | *Medicago lupulina* | **HM850164.1** AY395551.1 | **HM851136.1** **HM159569.1** |
| Fabales | Fabaceae | *Medicago polymorpha* | HM850165.1 | HM851137.1 AF522104.2 |
| Fabales | Fabaceae | *Ononis spinosa* | Z70174.1 |  |
| Fabales | Fabaceae | *Ornithopus perpusillus* | **HM850216.1** | **HM851143.1** |
| Fabales | Fabaceae | *Trifolium arvense* | **HM850409.1** | **HM851149.1** |
| Fabales | Fabaceae | *Trifolium campestre* | HM850410.1 | HM851150.1 |
| Fabales | Fabaceae | *Trifolium dubium* | HM850412.1 | HM851151.1 AF522121.1 FJ395427.1 |
| Fabales | Fabaceae | *Trifolium fragiferum* | **HM850413.1** | **HM851152.1** **AF522122.1** |
| Fabales | Fabaceae | *Trifolium ornithopodioides* | HM850418.1 | **HM851156.1** |
| Fabales | Fabaceae | *Trifolium pratense* | **HE574638.1** **HE574637.1** **HE574596.1** **HM850419.1** **AY395564.1** **HQ619749.1** **EU677058.1** **EU677057.1** **EU677056.1** **FJ395579.1** | **HM851157.1** **HQ619811.1** **EU749449.1** **EU749448.1** **EU749447.1** FJ395384.1 |
| Fabales | Fabaceae | *Trifolium repens* | **HM850420.1** **GQ436346.1** **EU677061.1** **EU677060.1** **EU677059.1** **FJ395599.1** | **HM851158.1** **AF522131.1** **EU749452.1** **EU749451.1** **EU749450.1** |
| Fabales | Fabaceae | *Trifolium scabrum* | **HM850422.1** | **HM851160.1** |
| Fabales | Fabaceae | *Trifolium squamosum* | HM850423.1 | **HM851161.1** |
| Fabales | Fabaceae | *Trifolium striatum* | **HM850425.1** | **HM851162.1** **AF522134.1** |
| Fabales | Fabaceae | *Trifolium subterraneum* | **HM850426.1** | **HM851163.1** **AF522135.1** |
| Fabales | Fabaceae | *Trifolium suffocatum* | HM850427.1 | HM851164.1 |
| Fabales | Fabaceae | *Ulex europaeus* | HM850431.1 Z70111.1 | HM851132.1 |
| Fabales | Fabaceae | *Vicia bithynica* | **HM850458.1** | **HM851167.1** |
| Fabales | Fabaceae | *Vicia cracca* | **HE574599.1** **HE574598.1** **HE574597.1** **AY395566.1** |  |
| Fabales | Fabaceae | *Vicia hirsuta* | **HM850459.1** | **HM851168.1** **AF522157.1** **HM026382.1** |
| Fabales | Fabaceae | *Vicia sativa* | **HM850461.1** **HM850456.1** **AB517630.1** |  |
| Fabales | Fabaceae | *Vicia sepium* | **FJ395590.1** |  |
| Fabales | Fabaceae | *Vicia tetrasperma* | **HM850463.1** **FJ395595.1** | **HM851171.1** **HM026384.1** |
| Fabales | Polygalaceae | *Polygala serpyllifolia* | **HM850272.1** **EU644685.1** |  |
| Fabales | Polygalaceae | *Polygala vulgaris* | **AM234193.1** **AJ829703.1** | EU604046.1 |
| Fagales | Betulaceae | *Alnus glutinosa* | **FJ844574.1** **HE574601.1** **HE574600.1** **EU644678.1** FN689372.1 | **FJ844593.1** **HQ600562.1** **FJ011816.1** |
| Fagales | Betulaceae | *Betula pendula* | GQ248556.1 GU373385.1 GU373382.1 | GQ248087.1 AY372014.1 AY372013.1 AY372012.1 AY372011.1 AY372010.1 AY372009.1 AY372008.1 FJ011829.1 GU373403.1 GU373400.1 AM889694.1 AM503811.1 AM503810.1 |
| Fagales | Betulaceae | *Betula pubescens* | GU373381.1 | AY372025.1 GU373399.1 |
| Fagales | Betulaceae | *Carpinus betulus* | **AY263928.1** | **FJ011781.1** **AY263915.1** **AF297392.1** **AY212015.1** **AJ417515.1** **AJ417514.1** **AJ417513.1** **AJ417512.1** **AJ417511.1** |
| Fagales | Betulaceae | *Corylus avellana* | **HM849918.1** **FR865138.1** FN689371.1 **AY263929.1** | **HM850898.1** **FR865055.1** **AY373445.1** **AY373444.1** **AY373443.1** **AY373442.1** **AY373441.1** **AY373440.1** **AY373439.1** **FJ011796.1** **AY263916.1** **AF297381.1** **AF297378.1** **AF297365.1** **AF297364.1** **AF297363.1** |
| Fagales | Fagaceae | *Castanea sativa* | **HM849869.1** **FN689363.1** **AF500363.1** | **HM850888.1** **AY042423.1** **AY042422.1** **AY042421.1** |
| Fagales | Fagaceae | *Fagus sylvatica* | **FN689362.1** **L13340.2** | **AB046507.1** |
| Fagales | Fagaceae | *Quercus petraea* | HQ619752.1 FN675740.1 FN675739.1 FN675738.1 AB125024.1 | HQ619814.1 FN675328.1 FN675327.1 FN675321.1 AY042472.1 AB125041.1 |
| Fagales | Fagaceae | *Quercus robur* | HQ619750.1 FN675735.1 FN675734.1 FN675733.1 AB125025.1 | HQ619812.1 FN675324.1 FN675323.1 FN675319.1 FJ185056.1 AY042488.1 AY042487.1 AY042468.1 FJ395434.1 AB125042.1 AJ491718.1 |
| Fagales | Myricaceae | *Myrica gale* | **X69530.1** **AJ626757.1** | **AY191715.1** |
| Gentianales | Apocynaceae | *Vinca minor* | **HQ619728.1** **AJ419768.1** | **DQ660553.1** **HQ619792.1** **AM295076.1** |
| Gentianales | Gentianaceae | *Blackstonia perfoliata* | **HM849816.1** |  |
| Gentianales | Gentianaceae | *Centaurium erythraea* | **HM849874.1** FJ395560.1 | **HM850820.1** FJ395364.1 |
| Gentianales | Gentianaceae | *Centaurium pulchellum* | HM849876.1 |  |
| Gentianales | Gentianaceae | *Centaurium scilloides* | HM849877.1 | HM850822.1 |
| Gentianales | Gentianaceae | *Cicendia filiformis* | **HM849894.1** |  |
| Gentianales | Gentianaceae | *Gentianella amarella* |  | AJ406355.1 AJ406326.1 |
| Gentianales | Rubiaceae | *Galium aparine* | **DQ006124.1** **HM850018.1** **X81091.1** | **HM850825.1** |
| Gentianales | Rubiaceae | *Galium mollugo* | HM850019.1 AY395538.1 HQ619780.1 FJ395593.1 | **HM850826.1** **HQ619836.1** **FJ395417.1** **AM503814.2** |
| Gentianales | Rubiaceae | *Galium palustre* | HM850020.1 **X81101.1** | **HM850827.1** |
| Gentianales | Rubiaceae | *Galium saxatile* | HM850022.1 | **HM850829.1** |
| Gentianales | Rubiaceae | *Sherardia arvensis* | **HM850351.1** **X81106.1** | **HM850831.1** |
| Geraniales | Geraniaceae | *Erodium moschatum* | **HM849983.1** |  |
| Geraniales | Geraniaceae | *Geranium dissectum* | HM850025.1 | **HM850907.1** **FJ395400.1** |
| Geraniales | Geraniaceae | *Geranium molle* | HM850026.1 | HM850908.1 FJ395361.1 |
| Geraniales | Geraniaceae | *Geranium purpureum* | HM850027.1 | HM850909.1 |
| Geraniales | Geraniaceae | *Geranium robertianum* |  | **FJ395401.1** |
| Geraniales | Geraniaceae | *Geranium rotundifolium* | HM850028.1 |  |
| Geraniales | Geraniaceae | *Geranium sanguineum* | FR865117.1 |  |
| Lamiales | Lamiaceae | *Ajuga reptans* | **U32163.1** **Z37385.1** | **AY840130.1** |
| Lamiales | Lamiaceae | *Ballota nigra* | HM849806.1 |  |
| Lamiales | Lamiaceae | *Clinopodium acinos* |  | **GU381664.1** **AY840144.1** |
| Lamiales | Lamiaceae | *Clinopodium vulgare* | **HM849904.1** | **HM850808.1** **AY840153.1** |
| Lamiales | Lamiaceae | *Glechoma hederacea* | **DQ006100.1** **HM850031.1** **AB266226.1** **AY570384.1** **L14292.1** **Z37391.1** | **HM850789.1** **AY840143.1** **FJ395424.1** |
| Lamiales | Lamiaceae | *Lamiastrum galeobdolon* | **HM850102.1** **Z37400.1** | **HM850791.1** |
| Lamiales | Lamiaceae | *Lamium album* | **HE574587.1** **FJ395588.1** **Z37398.1** **Z37397.1** **Z37396.1** | **FJ395404.1** **AJ429332.1** |
| Lamiales | Lamiaceae | *Lamium amplexicaule* | **HM850101.1** **AB266225.1** **Z37399.1** | **HM850790.1** |
| Lamiales | Lamiaceae | *Lamium purpureum* | HM850103.1 HQ619755.1 AB266224.1 U75702.1 Z37403.1 | HM850792.1 HQ619816.1 AM503816.2 |
| Lamiales | Lamiaceae | *Lycopus europaeus* | **HM850150.1** | **HM850793.1** **AY840154.1** |
| Lamiales | Lamiaceae | *Marrubium vulgare* | **HM850159.1** **HM590056.1** **U28875.1** **Z37412.1** **Z37411.1** | **HM850794.1** |
| Lamiales | Lamiaceae | *Mentha aquatica* |  | HM850796.1 FR719059.1 FR719058.1 |
| Lamiales | Lamiaceae | *Mentha pulegium* | HM850171.1 | HM850797.1 GU381687.1 |
| Lamiales | Lamiaceae | *Mentha spicata* | FR720535.1 FR720534.1 | FR719061.1 FR719060.1 GU381684.1 |
| Lamiales | Lamiaceae | *Mentha suaveolens* | HM850172.1 U28876.1 Z37417.1 | HM850798.1 GU381685.1 |
| Lamiales | Lamiaceae | *Nepeta cataria* | HM590057.1 **Z37421.1** |  |
| Lamiales | Lamiaceae | *Origanum vulgare* | **FR865126.1** **FR720565.1** **FR720563.1** **FR720562.1** FR720561.1 FR720560.1 **FR720559.1** **FJ395564.1** Z37427.1 | **HM850800.1** **FR865048.1** **FR719090.1** **FR719088.1** **FR719087.1** **FR719086.1** **FR719085.1** **FR719084.1** **GU381802.1** **AY840165.1** **FJ395365.1** |
| Lamiales | Lamiaceae | *Prunella vulgaris* | **HM850288.1** **AY395556.1** FJ513157.1 **Z37433.1** | **HM850805.1** **FJ395426.1** FJ513166.1 |
| Lamiales | Lamiaceae | *Salvia pratensis* | **AY570436.1** Z37447.1 |  |
| Lamiales | Lamiaceae | *Salvia verbenaca* | **HM850333.1** **AY570423.1** | **HM850803.1** |
| Lamiales | Lamiaceae | *Scutellaria galericulata* | Z37459.1 |  |
| Lamiales | Lamiaceae | *Scutellaria minor* | HM850345.1 | HM850804.1 |
| Lamiales | Lamiaceae | *Stachys arvensis* | **HM850384.1** | **HM850806.1** |
| Lamiales | Lamiaceae | *Stachys officinalis* | **AF502015.1** **Z37462.1** |  |
| Lamiales | Lamiaceae | *Stachys palustris* | HE574636.1 |  |
| Lamiales | Lamiaceae | *Stachys sylvatica* | AF502022.1 FJ395603.1 Z37464.1 | FJ395437.1 |
| Lamiales | Lamiaceae | *Teucrium scorodonia* | **HM850397.1** **Z37469.1** **Z37468.1** | **HM850801.1** |
| Lamiales | Lamiaceae | *Thymus pulegioides* |  | GU381790.1 |
| Lamiales | Lentibulariaceae | *Utricularia intermedia* |  | AF531839.1 |
| Lamiales | Lentibulariaceae | *Utricularia vulgaris* |  | **AF531831.1** |
| Lamiales | Oleaceae | *Fraxinus excelsior* | **FJ395592.1** **FJ862056.1** | **AM933427.1** **HM171528.1** **HM171524.1** **HM171522.1** **HM171489.1** **FJ395414.1** |
| Lamiales | Oleaceae | *Ligustrum vulgare* | **HQ619759.1** **L11686.1** **DQ673302.1** **FJ395605.1** **FJ862059.1** | **HQ619820.1** **AM933428.1** **FN668807.1** **FJ395441.1** |
| Lamiales | Orobanchaceae | *Lathraea squamaria* |  | **HM193524.1** **AM503817.1** |
| Lamiales | Orobanchaceae | *Melampyrum pratense* | **AM503852.2** X83721.1 | **AM503823.1** |
| Lamiales | Orobanchaceae | *Melampyrum sylvaticum* | **AM503854.2** | **AF051991.1** **AM503825.1** |
| Lamiales | Orobanchaceae | *Odontites vernus* | **FJ395565.1** | **FJ395366.1** |
| Lamiales | Orobanchaceae | *Orobanche hederae* |  | HM854939.1 AF051995.1 AJ429338.1 |
| Lamiales | Orobanchaceae | *Orobanche minor* |  | **HM854938.1** |
| Lamiales | Orobanchaceae | *Parentucellia viscosa* | **HM850234.1** **AY849865.1** | **HM850810.1** **HM193525.1** **AY849606.1** |
| Lamiales | Orobanchaceae | *Pedicularis sylvatica* |  | **AF531781.1** |
| Lamiales | Orobanchaceae | *Rhinanthus minor* | **AY395558.1** |  |
| Lamiales | Plantaginaceae | *Callitriche brutia* | HM849837.1 AF248009.1 |  |
| Lamiales | Plantaginaceae | *Callitriche hamulata* | AF248013.1 |  |
| Lamiales | Plantaginaceae | *Callitriche hermaphroditica* | **L36441.1** **AF248014.1** |  |
| Lamiales | Plantaginaceae | *Callitriche platycarpa* | AF248022.1 |  |
| Lamiales | Plantaginaceae | *Callitriche stagnalis* | HM849839.1 AF248023.1 | **HM851032.1** |
| Lamiales | Plantaginaceae | *Callitriche truncata* | **AF248025.1** |  |
| Lamiales | Plantaginaceae | *Digitalis purpurea* | **HM849951.1** **L01902.2** **X83720.1** | **HM850957.1** |
| Lamiales | Plantaginaceae | *Hippuris vulgaris* | **L36443.1** **AF248028.1** |  |
| Lamiales | Plantaginaceae | *Kickxia elatine* | **HM850096.1** | **HM850969.1** |
| Lamiales | Plantaginaceae | *Kickxia spuria* | HM850097.1 | HM850970.1 |
| Lamiales | Plantaginaceae | *Linaria repens* |  | **JF694193.1** |
| Lamiales | Plantaginaceae | *Linaria vulgaris* | **GQ248631.1** | JF694194.1 **AM889720.1** |
| Lamiales | Plantaginaceae | *Littorella uniflora* | **HM850128.1** | **HM851045.1** **FN773546.1** |
| Lamiales | Plantaginaceae | *Misopates orontium* | **HM850180.1** | **HM850960.1** **EU718055.1** |
| Lamiales | Plantaginaceae | *Plantago coronopus* | **HM850263.1** **HQ593827.1** **HQ593826.1** **HQ593825.1** **HQ593824.1** **AJ389600.1** | **HM851041.1** |
| Lamiales | Plantaginaceae | *Plantago lanceolata* | **HM850265.1** **GQ248673.1** **GQ248672.1** **L36454.1** **EU676932.1** **EU676931.1** **EU676930.1** | **HM851043.1** **GQ248179.1** **GQ248178.1** **EU749325.1** **EU749324.1** **EU749323.1** **EU718056.1** **FJ395430.1** |
| Lamiales | Plantaginaceae | *Plantago major* | **HM850266.1** **HQ619769.1** **GQ248674.1** **EU676935.1** **EU676934.1** **EU676933.1** FM207430.1 | **HM851044.1** **HQ619825.1** **GQ248180.1** **EU749328.1** **EU749327.1** **EU749326.1** |
| Lamiales | Plantaginaceae | *Plantago maritima* | **HQ593829.1** |  |
| Lamiales | Plantaginaceae | *Plantago media* | **AJ389596.1** | **AY667474.1** |
| Lamiales | Plantaginaceae | *Sibthorpia europaea* | **HM850352.1** | **HM850967.1** |
| Lamiales | Plantaginaceae | *Veronica agrestis* | **HM850446.1** **AJ247611.1** | HM851033.1 |
| Lamiales | Plantaginaceae | *Veronica anagallis* | AY034021.1 |  |
| Lamiales | Plantaginaceae | *Veronica arvensis* | **HM850447.1** **HQ619782.1** | **HM851034.1** **HQ619837.1** **AF052003.1** |
| Lamiales | Plantaginaceae | *Veronica catenata* | HM850448.1 | HM851035.1 |
| Lamiales | Plantaginaceae | *Veronica chamaedrys* | **FJ395608.1** **FJ395581.1** | **FJ395446.1** **FJ395387.1** |
| Lamiales | Plantaginaceae | *Veronica officinalis* | **HM850450.1** **AY034024.1** | **HM851037.1** |
| Lamiales | Plantaginaceae | *Veronica serpyllifolia* | **HM850454.1** | **HM851040.1** |
| Lamiales | Scrophulariaceae | *Limosella aquatica* |  | FN773544.1 |
| Lamiales | Scrophulariaceae | *Scrophularia auriculata* | **HM850343.1** |  |
| Lamiales | Scrophulariaceae | *Verbascum thapsus* | HM850441.1 L36452.1 | **HM850965.1** **AF052002.1** |
| Lamiales | Verbenaceae | *Verbena officinalis* | **HM850444.1** **GQ436523.1** **Z37473.1** | **HM850974.1** **HM853866.1** **GQ434147.1** **GQ434146.1** |
| Liliales | Colchicaceae | *Colchicum autumnale* | **FR865158.1** | **FR865065.1** **FN668829.1** |
| Liliales | Liliaceae | *Gagea bohemica* |  | **EU912103.1** |
| Liliales | Liliaceae | *Gagea lutea* | **AB034752.1** |  |
| Malpighiales | Elatinaceae | *Elatine hexandra* | HM849969.1 |  |
| Malpighiales | Elatinaceae | *Elatine hydropiper* | AJ402948.1 |  |
| Malpighiales | Euphorbiaceae | *Euphorbia exigua* | **HM849987.1** | **HM850916.1** |
| Malpighiales | Euphorbiaceae | *Euphorbia helioscopia* | **HM849988.1** **GU441790.1** **GU441789.1** | **HM850920.1** **GU441806.1** **GU441805.1** **EU659776.1** **EU659775.1** |
| Malpighiales | Euphorbiaceae | *Euphorbia lathyris* | HM849989.1 | **GQ434084.1** |
| Malpighiales | Euphorbiaceae | *Euphorbia peplis* | **HM849992.1** |  |
| Malpighiales | Euphorbiaceae | *Euphorbia peplus* | HM849993.1 | **HM850919.1** |
| Malpighiales | Euphorbiaceae | *Mercurialis annua* | HM850173.1 **X69745.1** | **HM850922.1** **DQ536104.1** **DQ536103.1** **AY918216.1** **AY918215.1** **AY918214.1** **AY918213.1** **AY918212.1** **AY918211.1** **AY918210.1** **AY918209.1** **AY918208.1** |
| Malpighiales | Euphorbiaceae | *Mercurialis perennis* | **AY794944.1** **FR865155.1** **FJ395570.1** | **FR865063.1** **DQ536113.1** **DQ536112.1** |
| Malpighiales | Hypericaceae | *Hypericum androsaemum* | **HQ332070.1** |  |
| Malpighiales | Hypericaceae | *Hypericum elodes* | **HM850060.1** **HQ332073.1** |  |
| Malpighiales | Hypericaceae | *Hypericum humifusum* | **HM850063.1** | HM850931.1 |
| Malpighiales | Hypericaceae | *Hypericum perforatum* | **HM850066.1** **HQ332081.1** AF206779.1 **GQ436684.1** **FJ395585.1** |  |
| Malpighiales | Hypericaceae | *Hypericum tetrapterum* | HQ332082.1 | HQ331631.1 |
| Malpighiales | Hypericaceae | *Hypericum undulatum* | HM850067.1 |  |
| Malpighiales | Linaceae | *Linum bienne* | **HM850126.1** **HM544063.1** **FJ169568.1** | **HM850982.1** **HM544102.1** |
| Malpighiales | Linaceae | *Linum catharticum* | **HM544064.1** **FJ169570.1** | **HM544103.1** |
| Malpighiales | Linaceae | *Radiola linoides* | HM850292.1 **HM544074.1** **FJ169598.1** | **HM850983.1** **HM544118.1** |
| Malpighiales | Salicaceae | *Populus nigra* | HM850278.1 JF429910.1 HQ619781.1 FN689367.1 AJ418828.1 | **HM850926.1** **JF429914.1** **AB038186.1** |
| Malpighiales | Salicaceae | *Populus tremula* | HE574617.1 JF429908.1 GQ248681.1 AJ418827.1 | **JF429912.1** **GQ248185.1** **AM889740.1** **AJ506088.1** **AJ506087.1** **AJ506086.1** **AJ506085.1** **AJ506084.1** **AJ506083.1** **AJ506082.1** |
| Malpighiales | Salicaceae | *Salix alba* | FN689366.1 GU373328.1 GU373327.1 GU373326.1 GU373325.1 GU373324.1 AJ849579.1 AJ849572.1 AB012780.1 | EU790677.1 GU373360.1 AJ849595.1 AJ849588.1 AJ849587.1 AJ849580.1 |
| Malpighiales | Salicaceae | *Salix aurita* | GU373329.1 |  |
| Malpighiales | Salicaceae | *Salix caprea* | FR694871.1 GU373333.1 GU373332.1 GU373331.1 GU373330.1 | FR695023.1 |
| Malpighiales | Salicaceae | *Salix cinerea* | GU373334.1 |  |
| Malpighiales | Salicaceae | *Salix fragilis* | GU373335.1 AJ849573.1 AJ418841.1 | AJ849589.1 AJ849581.1 |
| Malpighiales | Salicaceae | *Salix herbacea* | GU373338.1 | EU790671.1 EU790670.1 |
| Malpighiales | Salicaceae | *Salix pentandra* | GU373348.1 GU373326.1 GU373325.1 AJ849575.1 AB012791.1 | **EU790685.1** **EU790684.1** AJ849590.1 AJ849582.1 |
| Malpighiales | Salicaceae | *Salix purpurea* | GU373352.1 GU373351.1 AJ849578.1 | AY669057.1 AJ849592.1 AJ849584.1 |
| Malpighiales | Salicaceae | *Salix repens* | GU373355.1 GU373354.1 GU373353.1 GU373347.1 | GU373366.1 GU373365.1 GU373364.1 |
| Malpighiales | Salicaceae | *Salix triandra* | FJ788587.1 FN689365.1 **AJ849576.1** | **EU790687.1** **AJ849591.1** **AJ849585.1** |
| Malpighiales | Salicaceae | *Salix viminalis* | AJ849577.1 | AJ849594.1 AJ849586.1 |
| Malpighiales | Violaceae | *Viola hirta* | FR865127.1 | FR865049.1 |
| Malpighiales | Violaceae | *Viola odorata* | HM850467.1 | HM850936.1 |
| Malpighiales | Violaceae | *Viola palustris* | **HM850468.1** | **HM850937.1** |
| Malpighiales | Violaceae | *Viola reichenbachiana* | FR865134.1 |  |
| Malvales | Cistaceae | *Helianthemum nummularium* | **FR865140.1** |  |
| Malvales | Cistaceae | *Helianthemum oelandicum* | FJ492027.1 |  |
| Malvales | Cistaceae | *Tuberaria guttata* | **FJ225853.1** **AJ233120.1** |  |
| Malvales | Malvaceae | *Althaea officinalis* |  | **EU346765.1** |
| Malvales | Malvaceae | *Malva arborea* | HM850156.1 | HM850993.1 |
| Malvales | Malvaceae | *Malva moschata* |  | **EU346792.1** |
| Malvales | Malvaceae | *Malva neglecta* |  | EU346788.1 |
| Malvales | Malvaceae | *Malva sylvestris* | GQ248641.1 EF590548.1 | GQ248155.1 EF590410.1 EU346787.1 |
| Malvales | Thymelaeaceae | *Daphne laureola* | **HM849946.1** | **HM850899.1** |
| Malvales | Thymelaeaceae | *Daphne mezereum* | AF022132.1 **AJ297233.1** |  |
| Myrtales | Lythraceae | *Lythrum portula* | **HM850155.1** | **HM850988.1** |
| Myrtales | Lythraceae | *Lythrum salicaria* | **AF421496.1** |  |
| Myrtales | Onagraceae | *Chamerion angustifolium* | L10217.1 |  |
| Myrtales | Onagraceae | *Circaea alpina* | **L10216.1** |  |
| Myrtales | Onagraceae | *Circaea lutetiana* | **AM235666.1** |  |
| Myrtales | Onagraceae | *Epilobium hirsutum* | FJ395568.1 |  |
| Myrtales | Onagraceae | *Epilobium montanum* | FJ395563.1 |  |
| Myrtales | Onagraceae | *Epilobium obscurum* | HM849975.1 |  |
| Myrtales | Onagraceae | *Epilobium parviflorum* | HM849976.1 FJ395561.1 | **HM851001.1** |
| Myrtales | Onagraceae | *Epilobium tetragonum* | HM849977.1 FJ395557.1 | **HM851002.1** |
| Nymphaeales | Nymphaeaceae | *Nuphar lutea* | **DQ182338.1** | **DQ185532.1** **AF117100.1** **AF543741.1** |
| Nymphaeales | Nymphaeaceae | *Nymphaea alba* | **HM850203.1** **GQ358628.1** **GQ358627.1** | **HM850476.1** **GQ358630.1** **GQ358629.1** |
| Oxalidales | Oxalidaceae | *Oxalis acetosella* | **FJ670181.1** |  |
| Pinales | Cupressaceae | *Juniperus communis* | **HM024297.1** **HM024296.1** **HM024295.1** **AY988260.1** **HE574632.1** **HQ832767.1** **FR865157.1** **FR831943.1** **FR831939.1** **EU677075.1** **EU677074.1** **EU677073.1** **EU677072.1** **AY664859.1** |  |
| Pinales | Taxaceae | *Taxus baccata* | **HE574621.1** **FR831954.1** **FR831953.1** **FR831952.1** **HQ619788.1** **HM591068.1** **HM591067.1** **HM591066.1** **HM591065.1** **HM591064.1** **GQ248703.1** **EF590579.1** **EF660721.1** **AF456388.1** **FJ395607.1** **AJ235811.1** | **HQ619842.1** **HM591021.1** **HM591020.1** **HM591019.1** **HM591018.1** **HM591017.1** **AF457109.1** **DQ478791.1** **AB023996.1** |
| Poales | Cyperaceae | *Blysmus compressus* | **AM999788.1** **AJ404700.1** |  |
| Poales | Cyperaceae | *Bolboschoenus maritimus* | **JF313185.1** **HM849817.1** **AM999789.1** **Y12996.1** | **HM850837.1** |
| Poales | Cyperaceae | *Carex aquatilis* | FJ548247.1 FJ548246.1 FJ904603.1 FJ904602.1 FJ904601.1 FJ904600.1 FJ904599.1 FJ904598.1 FJ904597.1 FJ904596.1 FJ904595.1 FJ904594.1 |  |
| Poales | Cyperaceae | *Carex bigelowii* | FJ548252.1 FJ548251.1 | FJ548083.1 FJ548082.1 FJ548081.1 FJ548080.1 FJ548079.1 FJ548078.1 FJ548077.1 |
| Poales | Cyperaceae | *Carex capillaris* | FJ548255.1 FJ548254.1 FJ548253.1 |  |
| Poales | Cyperaceae | *Carex distans* | **GQ469848.1** |  |
| Poales | Cyperaceae | *Carex divulsa* | HM849851.1 | **HM850840.1** |
| Poales | Cyperaceae | *Carex echinata* | HM849852.1 |  |
| Poales | Cyperaceae | *Carex extensa* | **HM849853.1** **GQ469846.1** | **HM850844.1** |
| Poales | Cyperaceae | *Carex hostiana* | L12672.2 GQ469841.1 |  |
| Poales | Cyperaceae | *Carex magellanica* | GQ469849.1 |  |
| Poales | Cyperaceae | *Carex muricata* | GQ248561.2 |  |
| Poales | Cyperaceae | *Carex nigra* | HM849856.1 AY395533.1 GQ469838.1 | HM850842.1 FN668463.1 |
| Poales | Cyperaceae | *Carex otrubae* | HM849857.1 | **HM850843.1** |
| Poales | Cyperaceae | *Carex ovalis* | **HM849858.1** | **HM850845.1** |
| Poales | Cyperaceae | *Carex panicea* | **HM849860.1** |  |
| Poales | Cyperaceae | *Carex pauciflora* | **GQ469850.1** |  |
| Poales | Cyperaceae | *Carex pendula* | **HM849861.1** **AM999796.1** | **HM850847.1** |
| Poales | Cyperaceae | *Carex pilulifera* | HM849863.1 | HM850849.1 |
| Poales | Cyperaceae | *Carex pulicaris* | **GQ469843.1** |  |
| Poales | Cyperaceae | *Carex punctata* | HM849864.1 |  |
| Poales | Cyperaceae | *Carex rostrata* | GQ469851.1 |  |
| Poales | Cyperaceae | *Carex viridula* | HM849865.1 | **HM850850.1** |
| Poales | Cyperaceae | *Cladium mariscus* | **HM849902.1** **AM999799.1** **DQ058338.1** | **HM850838.1** |
| Poales | Cyperaceae | *Cyperus longus* | **HM849940.1** **AM999810.1** **Y13015.1** | **HM850855.1** |
| Poales | Cyperaceae | *Eleocharis acicularis* | **GU344675.1** **AM999819.1** |  |
| Poales | Cyperaceae | *Eleocharis multicaulis* | **HM849970.1** | **HM850942.1** |
| Poales | Cyperaceae | *Eleocharis palustris* | **HM849971.1** **AM999826.1** | **HM850943.1** |
| Poales | Cyperaceae | *Eleocharis quinqueflora* | U49229.1 Y13010.1 |  |
| Poales | Cyperaceae | *Eriophorum vaginatum* | AM999830.1 Y12951.1 |  |
| Poales | Cyperaceae | *Isolepis cernua* | **HM850076.1** **AM999840.1** **Y13014.1** | **HM850939.1** |
| Poales | Cyperaceae | *Isolepis setacea* | **HM850078.1** **Y12962.1** | HM850941.1 |
| Poales | Cyperaceae | *Rhynchospora alba* | **AM999856.1** |  |
| Poales | Cyperaceae | *Schoenoplectus lacustris* | **AM999865.1** Y12943.1 |  |
| Poales | Cyperaceae | *Schoenoplectus tabernaemontani* | **GQ130365.1** |  |
| Poales | Cyperaceae | *Schoenus nigricans* | **AM999870.1** **Y12983.1** |  |
| Poales | Cyperaceae | *Scirpus sylvaticus* | **EF178586.1** **AM999875.1** |  |
| Poales | Cyperaceae | *Trichophorum cespitosum* | Y12969.1 AJ811004.1 AJ811003.1 |  |
| Poales | Juncaceae | *Juncus acutus* | HM850083.1 **AM999842.1** | **HM850944.1** |
| Poales | Juncaceae | *Juncus articulatus* | HM850084.1 AY395543.1 AY216614.1 | HM850945.1 |
| Poales | Juncaceae | *Juncus bufonius* | **HM850085.1** **AY216615.1** | **HM850947.1** |
| Poales | Juncaceae | *Juncus bulbosus* | HM850086.1 AY216622.1 | HM850946.1 AY973524.1 |
| Poales | Juncaceae | *Juncus capitatus* | HM850087.1 |  |
| Poales | Juncaceae | *Juncus compressus* | **AY216625.1** |  |
| Poales | Juncaceae | *Juncus conglomeratus* | HM850088.1 | **HM850949.1** |
| Poales | Juncaceae | *Juncus effusus* | HM850089.1 AY216612.1 L12681.1 | **HM850950.1** **AY973527.1** **AB088803.1** |
| Poales | Juncaceae | *Juncus gerardii* | AY216613.1 |  |
| Poales | Juncaceae | *Juncus maritimus* | **HM850090.1** **AY216629.1** | **HM850951.1** |
| Poales | Juncaceae | *Juncus squarrosus* | **AY216619.1** |  |
| Poales | Juncaceae | *Juncus subnodulosus* | **AY216630.1** |  |
| Poales | Juncaceae | *Juncus triglumis* | **AY216605.1** |  |
| Poales | Juncaceae | *Luzula campestris* | HM850146.1 AY395548.1 AY216652.1 | HM850953.1 FJ395407.1 |
| Poales | Juncaceae | *Luzula multiflora* | HM850148.1 AJ419945.1 | HM850955.1 |
| Poales | Juncaceae | *Luzula pilosa* | **AY216653.1** |  |
| Poales | Juncaceae | *Luzula sylvatica* | **AY216637.1** |  |
| Poales | Poaceae | *Agrostis canina* |  | FJ231115.1 FJ231114.1 FJ231113.1 FJ231112.1 DQ146797.1 |
| Poales | Poaceae | *Agrostis capillaris* | **AY395527.1** | **FJ231112.1** **DQ146798.1** **FJ395420.1** **AM234560.1** |
| Poales | Poaceae | *Agrostis gigantea* |  | DQ146814.1 DQ146803.1 DQ146802.1 |
| Poales | Poaceae | *Agrostis stolonifera* | HM849748.1 AJ746280.1 | HM850556.1 FJ231115.1 FJ231114.1 FJ231113.1 FJ231112.1 DQ146826.1 DQ146825.1 DQ146824.1 DQ146823.1 DQ146822.1 DQ146821.1 DQ146820.1 DQ146819.1 DQ146818.1 DQ146817.1 DQ146816.1 DQ146815.1 DQ146814.1 DQ146813.1 DQ146812.1 |
| Poales | Poaceae | *Agrostis vinealis* |  | FJ231113.1 DQ146813.1 |
| Poales | Poaceae | *Aira caryophyllea* | HM849751.1 AM849361.1 | **HM850557.1** **DQ786878.1** |
| Poales | Poaceae | *Aira praecox* | **HM849752.1** **AJ746255.1** | **HM850558.1** **AM234540.1** |
| Poales | Poaceae | *Alopecurus geniculatus* | HM849758.1 AJ746281.1 | **HM850563.1** |
| Poales | Poaceae | *Alopecurus pratensis* | **EF125141.1** **HM849759.1** **AY395528.1** **AJ784837.1** | **HM850564.1** **EU434293.1** **FJ395391.1** |
| Poales | Poaceae | *Ammophila arenaria* |  | AM234561.1 |
| Poales | Poaceae | *Anisantha sterilis* | **HQ619771.1** **AY836155.1** | **HM770812.1** **HQ619827.1** |
| Poales | Poaceae | *Anthoxanthum odoratum* | **HM849780.1** **AJ746282.1** **AJ746256.1** | **HM850562.1** **DQ786884.1** **AM234541.1** |
| Poales | Poaceae | *Arrhenatherum elatius* | HM849793.1 **AY395529.1** **AJ784823.1** | **HM850561.1** **EU434292.1** **FJ395406.1** **AM234543.1** |
| Poales | Poaceae | *Avena fatua* | **HM849804.1** **AJ746257.1** | **HM850560.1** **GU367282.1** **GU367281.1** **GU367280.1** **EU833847.1** |
| Poales | Poaceae | *Avenula pubescens* |  | **FM957003.1** |
| Poales | Poaceae | *Brachypodium pinnatum* | AY632361.1 AM849347.1 | **DQ786891.1** |
| Poales | Poaceae | *Brachypodium sylvaticum* | HM849820.1 AJ746258.1 | HM850579.1 **FR694878.1** AF164400.1 |
| Poales | Poaceae | *Briza media* | FR865143.1 **AJ746285.1** | **FR865056.1** **AM234610.1** |
| Poales | Poaceae | *Bromopsis erecta* | FR865129.1 AJ746286.1 | **FR865051.1** **AM234570.1** |
| Poales | Poaceae | *Bromopsis ramosa* |  | **FJ395419.1** |
| Poales | Poaceae | *Bromus commutatus* | AJ746287.1 | FJ395418.1 |
| Poales | Poaceae | *Bromus hordeaceus* | HM849826.1 AY395531.1 GQ248557.1 | HM850582.1 AM889695.1 GQ248088.1 FJ395380.1 |
| Poales | Poaceae | *Bromus secalinus* |  | AM234571.1 |
| Poales | Poaceae | *Calamagrostis epigejos* | **AJ784820.1** |  |
| Poales | Poaceae | *Catabrosa aquatica* |  | **DQ786898.1** **AM234589.1** |
| Poales | Poaceae | *Catapodium marinum* |  | HM850565.1 |
| Poales | Poaceae | *Catapodium rigidum* | EF125150.1 HM849870.1 | HM850566.1 AM234586.1 |
| Poales | Poaceae | *Cynosurus cristatus* | **EF125151.1** **HM849932.1** | **HM850529.1** **DQ786901.1** |
| Poales | Poaceae | *Dactylis glomerata* | **HM849945.1** **FR865119.1** **AY395535.1** **AJ746268.1** | **HM850569.1** **FR865044.1** **DQ786902.1** **FJ395392.1** **AM234595.1** |
| Poales | Poaceae | *Danthonia decumbens* | **EU400662.1** | **EU400729.1** |
| Poales | Poaceae | *Deschampsia cespitosa* | EF125152.1 AY691635.1 | DQ786903.1 **FJ395402.1** **AM234546.1** |
| Poales | Poaceae | *Deschampsia flexuosa* |  | **DQ786887.1** **AM234545.1** |
| Poales | Poaceae | *Elytrigia repens* | HE574609.1 HM849973.1 | **HM850576.1** **FJ395421.1** |
| Poales | Poaceae | *Festuca altissima* |  | **AM234585.1** |
| Poales | Poaceae | *Festuca arundinacea* | HM850001.1 | **HM850531.1** **DQ786940.1** |
| Poales | Poaceae | *Festuca gigantea* |  | **AM234720.1** |
| Poales | Poaceae | *Festuca ovina* |  | FJ395397.1 |
| Poales | Poaceae | *Festuca pratensis* | HM850003.1 AY395536.1 | HM850535.1 |
| Poales | Poaceae | *Gastridium ventricosum* |  | DQ786914.1 |
| Poales | Poaceae | *Gaudinia fragilis* | **EF125143.1** **HM850023.1** | **HM850536.1** **DQ786915.1** |
| Poales | Poaceae | *Glyceria declinata* | HM850032.1 | **HM850537.1** |
| Poales | Poaceae | *Glyceria fluitans* | HM850033.1 AY395539.1 AJ746290.1 | HM850538.1 |
| Poales | Poaceae | *Holcus lanatus* | **HM850053.1** **AM235062.1** **AJ746279.1** | **HM850540.1** **FJ395394.1** |
| Poales | Poaceae | *Holcus mollis* |  | **AM234554.1** |
| Poales | Poaceae | *Hordelymus europaeus* | **EF125163.1** **EU376159.1** **EU376158.1** **EU376157.1** **EU376156.1** **EU376155.1** **EU376154.1** **EU376153.1** | AM234596.1 |
| Poales | Poaceae | *Hordeum marinum* | HM850055.1 **AY137436.1** AY137434.1 AY836171.1 AY137433.1 AY137432.1 AY137431.1 AY137430.1 AY137429.1 AY137428.1 AY137427.1 AY137426.1 AY137425.1 AY137424.1 AY137423.1 **AY137422.1** **AY137421.1** **AY137420.1** **AY137419.1** **AY137418.1** | HM850542.1 FR694880.1 FJ897873.1 FJ897872.1 EU118398.1 EU118397.1 **EU118396.1** |
| Poales | Poaceae | *Hordeum murinum* | HM850056.1 **AY137437.1** **AY836172.1** | **HM850543.1** **EU118380.1** **EU118379.1** **EU118378.1** **EU118377.1** |
| Poales | Poaceae | *Hordeum secalinum* | AY395541.1 **AY601672.1** AJ746292.1 | **EU118399.1** |
| Poales | Poaceae | *Lolium perenne* | HM850132.1 AY395547.1 AJ746293.1 | **HM850533.1** **DQ786925.1** EU434291.1 **FJ395431.1** |
| Poales | Poaceae | *Melica uniflora* | **AJ746294.1** **AJ746263.1** |  |
| Poales | Poaceae | *Mibora minima* |  | **FR694894.1** **DQ786927.1** |
| Poales | Poaceae | *Milium effusum* |  | **AM234598.1** |
| Poales | Poaceae | *Molinia caerulea* | **AY632367.1** **AJ746295.1** | **AF164411.1** |
| Poales | Poaceae | *Nardus stricta* | **HM850196.1** **AJ746296.1** | **HM850544.1** **EU434289.1** **AF164394.1** **AM234573.1** |
| Poales | Poaceae | *Parapholis incurva* | EF125154.1 | DQ786931.1 AM234583.1 |
| Poales | Poaceae | *Phalaris arundinacea* | **AJ784827.1** | **AF164396.1** |
| Poales | Poaceae | *Phleum bertolonii* |  | **FJ395422.1** |
| Poales | Poaceae | *Phleum pratense* | HM850252.1 AY395554.1 AJ784832.1 AJ746298.1 | **HM850515.1** **DQ786932.1** **AF164397.1** FJ395422.1 **FJ395368.1** |
| Poales | Poaceae | *Phragmites australis* | **U29900.1** **EF423005.1** **AY545890.1** **U13229.1** **AJ746299.1** | **EU873209.1** **EU873208.1** **EU873207.1** **EU873206.1** **EU873205.1** **EU873204.1** **EU873203.1** **EU873202.1** **EU873201.1** **EU873200.1** **EU873199.1** **EU873198.1** **EU873197.1** **EU873196.1** **EU873195.1** **EU873194.1** **EU873193.1** **EU873192.1** **EU732698.1** **EU732697.1** |
| Poales | Poaceae | *Poa alpina* |  | **DQ786933.1** |
| Poales | Poaceae | *Poa annua* | **HQ619772.1** **EU676939.1** **EU676938.1** | **HQ619828.1** **EU749332.1** **EU749331.1** **AM234593.1** |
| Poales | Poaceae | *Poa compressa* | EU676942.1 EU676941.1 EU676940.1 | **EU749335.1** **EU749334.1** **EU749333.1** |
| Poales | Poaceae | *Poa glauca* |  | FN668470.1 |
| Poales | Poaceae | *Poa pratensis* |  | HM850516.1 AF164402.1 FJ395415.1 |
| Poales | Poaceae | *Poa trivialis* | HM850270.1 AY395555.1 AJ746301.1 | **HM850517.1** **FJ395369.1** |
| Poales | Poaceae | *Puccinellia distans* | L14621.1 | **DQ786938.1** |
| Poales | Poaceae | *Sesleria caerulea* | **FR865131.1** **EF125156.1** | **FR865052.1** **DQ786942.1** |
| Poales | Poaceae | *Spartina anglica* | **AM849382.1** |  |
| Poales | Poaceae | *Trisetum flavescens* | **AY395565.1** **AJ746276.1** |  |
| Poales | Poaceae | *Vulpia bromoides* | HM850470.1 | **HM850527.1** **FJ395408.1** |
| Poales | Poaceae | *Vulpia ciliata* | EF125157.1 |  |
| Poales | Poaceae | *Vulpia myuros* |  | **AF164403.1** |
| Poales | Typhaceae | *Sparganium emersum* | GU344676.1 HE574620.1 |  |
| Poales | Typhaceae | *Typha angustifolia* | AY952434.1 GQ436382.1 GQ436381.1 EU677063.1 EU677062.1 AM110250.1 | AY952419.1 GQ434092.1 EU749454.1 EU749453.1 AM114723.1 |
| Poales | Typhaceae | *Typha latifolia* | L05464.1 EU677065.1 EU677064.1 DQ069503.1 M91634.1 | EU749456.1 EU749455.1 DQ069587.1 AB088801.1 |
| Ranunculales | Papaveraceae | *Chelidonium majus* | **HM849887.1** **HQ829855.1** **DQ912892.1** | **HM851025.1** **GQ434280.1** |
| Ranunculales | Papaveraceae | *Fumaria capreolata* | HM850014.1 | HM851022.1 |
| Ranunculales | Papaveraceae | *Fumaria muralis* | HM850015.1 | HM851023.1 |
| Ranunculales | Papaveraceae | *Glaucium flavum* | **U86626.1** |  |
| Ranunculales | Papaveraceae | *Papaver dubium* | **HM850229.1** **AB517147.1** | HM851026.1 |
| Ranunculales | Papaveraceae | *Papaver rhoeas* | HM850230.1 **DQ912900.1** **GQ248663.1** **FJ626614.1** **EF590555.1** | HM851027.1 **GQ248175.1** **FJ626525.1** |
| Ranunculales | Papaveraceae | *Papaver somniferum* | **HM850232.1** **HM850231.1** **DQ912894.1** | **HM851029.1** **HM851028.1** |
| Ranunculales | Ranunculaceae | *Aconitum napellus* | **EU053898.1** | **FN668831.1** |
| Ranunculales | Ranunculaceae | *Aquilegia vulgaris* | **HM849784.1** **FJ449851.1** **EU053905.1** |  |
| Ranunculales | Ranunculaceae | *Caltha palustris* | **AY395532.1** **EU053906.1** **L02431.2** **FJ626581.1** | **AY515232.1** **FJ626494.1** **FJ597997.1** **AB069845.1** |
| Ranunculales | Ranunculaceae | *Clematis vitalba* | **FR865151.1** | **FR865061.1** **AB110525.1** |
| Ranunculales | Ranunculaceae | *Helleborus viridis* | **HQ619774.1** | **HQ619830.1** **AJ414339.1** **AJ414338.1** |
| Ranunculales | Ranunculaceae | *Myosurus minimus* | **DQ099441.1** |  |
| Ranunculales | Ranunculaceae | *Ranunculus acris* | AY395557.1 | **AY954199.1** **FM242742.1** |
| Ranunculales | Ranunculaceae | *Ranunculus aquatilis* |  | FM242779.1 |
| Ranunculales | Ranunculaceae | *Ranunculus auricomus* | **HE574635.1** | **FM242739.1** **FJ625807.1** **FJ625806.1** **FJ625805.1** **FJ625804.1** **FJ625803.1** **FJ625802.1** **FJ625801.1** **FJ625800.1** **FJ625799.1** **FJ625798.1** **FJ625797.1** |
| Ranunculales | Ranunculaceae | *Ranunculus bulbosus* | HM850293.1 HQ619775.1 |  |
| Ranunculales | Ranunculaceae | *Ranunculus circinatus* | GU344677.1 |  |
| Ranunculales | Ranunculaceae | *Ranunculus ficaria* | **EU053919.1** | **AY954232.1** **AY437814.1** |
| Ranunculales | Ranunculaceae | *Ranunculus flammula* | HM850295.1 | **HM851059.1** **AY954204.1** |
| Ranunculales | Ranunculaceae | *Ranunculus lingua* |  | **AY954206.1** |
| Ranunculales | Ranunculaceae | *Ranunculus parviflorus* | HM850297.1 |  |
| Ranunculales | Ranunculaceae | *Ranunculus peltatus* |  | HQ894444.1 AY954131.1 |
| Ranunculales | Ranunculaceae | *Ranunculus repens* | HM850298.1 |  |
| Ranunculales | Ranunculaceae | *Ranunculus sceleratus* | **AB517148.1** | **GU257993.1** |
| Ranunculales | Ranunculaceae | *Ranunculus trichophyllus* | EU053922.1 L08766.1 | HQ894447.1 AY954133.1 |
| Ranunculales | Ranunculaceae | *Thalictrum minus* | EU053923.1 |  |
| Ranunculales | Ranunculaceae | *Trollius europaeus* | **HE574595.1** | **HQ440178.1** **AY515236.1** |
| Rosales | Cannabaceae | *Humulus lupulus* | **DQ006077.1** **AF206777.1** **FN689369.1** **AF061992.1** **AB033892.1** **AB033894.1** **AB033761.1** | **AY257528.1** **AF345318.1** |
| Rosales | Rhamnaceae | *Frangula alnus* | **EU676982.1** **EU676981.1** **AJ390026.1** | **EU749374.1** **EU749373.1** **AY257532.1** |
| Rosales | Rhamnaceae | *Rhamnus cathartica* | **EU676980.1** **EU676979.1** **EU676978.1** **EU676977.1** **L13189.2** **X69752.1** | **EU749372.1** **EU749371.1** **EU749370.1** **EU749369.1** **AY257533.1** |
| Rosales | Rosaceae | *Agrimonia eupatoria* | HM849744.1 | HM850683.1 FJ395410.1 |
| Rosales | Rosaceae | *Crataegus monogyna* | HQ619776.1 FN689370.1 | HQ619832.1 AF288099.1 FJ395386.1 |
| Rosales | Rosaceae | *Dryas octopetala* | **JF317483.1** | **JF317424.1** **DQ851225.1** |
| Rosales | Rosaceae | *Filipendula vulgaris* | HM850007.1 **JF317486.1** U06804.1 | **HM850685.1** |
| Rosales | Rosaceae | *Fragaria vesca* | **HE574629.1** **HM850009.1** **HQ619777.1** | **HM850686.1** **HQ619833.1** **AF288102.1** **EU025925.1** |
| Rosales | Rosaceae | *Malus sylvestris* |  | **AF309231.1** **AF309230.1** **AF309177.1** **AM042563.1** **AM042562.1** **AM042561.1** |
| Rosales | Rosaceae | *Potentilla anglica* | HM850284.1 | HM850687.1 |
| Rosales | Rosaceae | *Potentilla anserina* |  | **AF288113.1** |
| Rosales | Rosaceae | *Potentilla erecta* | HM850285.1 | HM850688.1 |
| Rosales | Rosaceae | *Potentilla reptans* | HM850287.1 FJ395596.1 | HM850690.1 FJ395425.1 |
| Rosales | Rosaceae | *Potentilla sterilis* |  | **FJ395447.1** |
| Rosales | Rosaceae | *Prunus avium* |  | **FN668843.1** FJ395442.1 **AM503828.1** |
| Rosales | Rosaceae | *Prunus cerasus* |  | **FN668844.1** |
| Rosales | Rosaceae | *Prunus domestica* | L01947.2 AF227903.1 AF227901.1 | FN668845.1 |
| Rosales | Rosaceae | *Prunus padus* | **AF411485.1** **GU363800.1** **GU363791.1** | **GU363752.1** |
| Rosales | Rosaceae | *Prunus spinosa* | **FR865159.1** **FN689384.1** AF227904.1 | FR865066.1 FJ395440.1 AB036620.1 |
| Rosales | Rosaceae | *Pyrus communis* |  | AF288120.1 DQ860473.1 |
| Rosales | Rosaceae | *Rosa arvensis* |  | AB048598.1 |
| Rosales | Rosaceae | *Rosa canina* | FN689381.1 | FJ395439.1 AB011980.1 |
| Rosales | Rosaceae | *Rosa pimpinellifolia* |  | **AB011976.1** AB039290.1 |
| Rosales | Rosaceae | *Rosa rubiginosa* |  | AB011981.1 |
| Rosales | Rosaceae | *Rubus caesius* | FN689382.1 |  |
| Rosales | Rosaceae | *Rubus chamaemorus* |  | **AY366358.1** |
| Rosales | Rosaceae | *Rubus idaeus* | **HE574618.1** **U06825.1** **EU676990.1** **EU676989.1** **EU676988.1** **EU676987.1** **EU676986.1** | **EU749382.1** **EU749381.1** **EU749380.1** **EU749379.1** **EU749378.1** |
| Rosales | Rosaceae | *Sanguisorba minor* | **HM850336.1** **FR865160.1** | **HM850691.1** **FR865067.1** **AB073694.1** |
| Rosales | Rosaceae | *Sanguisorba officinalis* | **AY395560.1** **GQ436571.1** **GQ436570.1** **GQ436569.1** **GQ436566.1** **GU363794.1** **GU363793.1** | **GQ434183.1** **GQ434182.1** **GQ434181.1** **GQ434178.1** **GU363757.1** **GU363756.1** **GU363755.1** **AB073696.1** |
| Rosales | Rosaceae | *Sorbus aria* | GQ248697.1 | GQ248202.1 AM889746.1 |
| Rosales | Rosaceae | *Sorbus rupicola* | GQ248698.1 | GQ248203.1 AM889747.1 |
| Rosales | Rosaceae | *Sorbus torminalis* |  | AF309229.1 AF309198.1 |
| Rosales | Ulmaceae | *Ulmus glabra* | HE574622.1 |  |
| Rosales | Ulmaceae | *Ulmus minor* | FN689364.1 |  |
| Rosales | Ulmaceae | *Ulmus procera* | **HM850433.1** | **HM851106.1** |
| Rosales | Urticaceae | *Parietaria judaica* | **HM850236.1** **FJ432248.1** |  |
| Rosales | Urticaceae | *Urtica dioica* | **AY208707.1** **AF500361.1** **FJ395600.1** | **FJ395433.1** **GU266610.1** |
| Rosales | Urticaceae | *Urtica urens* | **HM850437.1** **FJ432249.1** | HM851111.1 EU002192.1 |
| Santalales | Santalaceae | *Viscum album* | **L26078.1** |  |
| Sapindales | Sapindaceae | *Acer campestre* | **FN689361.1** **DQ978399.1** | **FJ395381.1** **AJ438792.1** **AJ438791.1** **AJ438790.1** **AJ438789.1** **AJ438796.1** **AJ438795.1** **AJ438794.1** **AJ438793.1** **AJ438787.1** **AJ438788.1** |
| Saxifragales | Crassulaceae | *Sedum telephium* |  | **AF115669.1** |
| Saxifragales | Crassulaceae | *Umbilicus rupestris* | **HM850435.1** | **HM850701.1** **AF115684.1** |
| Saxifragales | Haloragaceae | *Myriophyllum spicatum* | **GU135245.1** **GU344679.1** |  |
| Saxifragales | Haloragaceae | *Myriophyllum verticillatum* | **GU344678.1** |  |
| Saxifragales | Saxifragaceae | *Chrysosplenium alternifolium* |  | **AM396496.1** |
| Saxifragales | Saxifragaceae | *Saxifraga nivalis* |  | **AF115491.1** |
| Saxifragales | Saxifragaceae | *Saxifraga oppositifolia* | **U06217.1** |  |
| Saxifragales | Saxifragaceae | *Saxifraga stellaris* |  | **AF115493.1** |
| Solanales | Convolvulaceae | *Calystegia sepium* | AY100992.1 HM849841.1 FJ395604.1 | **HM850892.1** **FJ395438.1** |
| Solanales | Convolvulaceae | *Convolvulus arvensis* | **AY100993.1** **HM849909.1** **AY558864.1** **FJ395582.1** | **HM850893.1** **FJ395388.1** |
| Solanales | Convolvulaceae | *Cuscuta epithymum* | AY558866.1 |  |
| Solanales | Solanaceae | *Atropa belladonna* | **FJ914178.1** **HQ216117.1** **U08609.1** | **EF438839.1** **EF438821.1** **FN668828.1** **AJ585882.1** |
| Solanales | Solanaceae | *Hyoscyamus niger* | **HQ216125.1** **HQ216124.1** | **EF438829.1** |
| Solanales | Solanaceae | *Solanum dulcamara* | **HM850363.1** **EU677012.1** **EU677011.1** **EU677010.1** **FJ395606.1** | **HM851100.1** **FN668838.1** **EU749404.1** **EU749403.1** **EU749402.1** **FJ395444.1** |
| Solanales | Solanaceae | *Solanum nigrum* | **GQ436617.1** **HM850367.1** **EU677015.1** **EU677014.1** **EU677013.1** | **HM851098.1** **FN668839.1** **EU749407.1** **EU749406.1** **EU749405.1** |
